# Unified representation learning for spatial multi-omics

**DOI:** 10.1093/bioinformatics/btag629

**Published:** 2026-08-24

**Authors:** Haoxuan Li, Sijie Wan, Hongyu Zhang, Zheng Wang, Yuansong Zeng

**Affiliations:** School of Big Data and Software Engineering, Chongqing University, Chongqing, 401331, China; School of Big Data and Software Engineering, Chongqing University, Chongqing, 401331, China; School of Big Data and Software Engineering, Chongqing University, Chongqing, 401331, China; Jinfeng Laboratory, Chongqing, 400039, China; School of Big Data and Software Engineering, Chongqing University, Chongqing, 401331, China

## Abstract

**Motivation:**

Integrating spatial multi-omics data is crucial for decoding tissue function, yet existing methods struggle with noise and cross-modal alignment.

**Results:**

We introduce SpaAlign, a novel two-stage framework for the robust integration of multiple omics and histological data. At its core, a contrastive learning stage creates a unified semantic space, followed by a self-supervised clustering stage that enforces structural coherence. Our evaluations demonstrate its effectiveness in accurately delineating the capsule-cortex-medulla architecture in human lymph nodes and precisely identifying germinal centers in human tonsil data, outperforming baseline methods.

**Availability and Implementation:**

The source code is available at https://github.com/VitaIntelli-CQU/SpaAlign. The source code is archived at https://doi.org/10.5281/zenodo.20250508.

## 1 Introduction

Spatially resolved transcriptomics is a breakthrough technology that anchors gene expression within its native tissue microenvironment, offering unprecedented insights into cellular heterogeneity and tissue architecture. Whether sequencing-based [e.g. 10× Visium (Ji *et al.* 2020), Slide-seq (Rodriques *et al.* 2019)] or imaging-based [e.g. MERFISH (Chen *et al.* 2015), seqFISH+ (Shah *et al.* 2016)], its central contribution lies in the simultaneous capture of transcriptional profiles and spatial coordinates. However, the complexity of biological systems arises from intricate interactions between multiple molecular layers. Relying solely on single-modality transcriptomic data is therefore insufficient to achieve a comprehensive understanding of cellular activities, creating the pressing need to integrate additional molecular layers.

This need has catalyzed the rapid advancement of spatially resolved multi-omics, aiming to construct multilayered molecular atlases that integrate diverse data types such as the epigenome, proteome, and metabolome (Vicari *et al.* 2024). Joint analysis of these modalities, often in conjunction with a high-resolution hematoxylin and eosin (H&E)-stained histology image, provides an essential bridge between the molecular blueprint and the functional or pathological state of the tissue (Ben-Chetrit *et al.* 2023, Liao *et al.* 2023, Liu *et al.* 2023, Zhang *et al.* 2023, Coleman *et al.* 2025). However, the path to a truly comprehensive view is paved with significant computational challenges. The various data modalities exhibit large differences in their statistical distributions, dimensionality, and sparsity, and are all subject to considerable technical noise. Therefore, developing computational methods that can overcome this profound heterogeneity to achieve deep and robust integration has become the core prerequisite for transforming massive spatial multi-omics data into precise biological insights.

Prior computational methods for spatial omics can be broadly categorized into three classes, each with distinct limitations. Early spatial analysis frameworks such as STAGATE (Dong and Zhang 2022), GraphST (Long *et al.* 2023), PAST (Li *et al.* 2023), and MUSE (Bao *et al.* 2022) primarily focus on spatial representation learning from limited modalities and often lack the ability to comprehensively integrate heterogeneous molecular signals across multiple omics layers. In contrast, powerful non-spatial integration frameworks such as totalVI (Gayoso *et al.* 2021), MultiVI (Ashuach *et al.* 2023), and GLUE (Cao and Gao 2022) effectively align multiple molecular modalities but do not explicitly model spatial coordinates or tissue architecture, limiting their applicability to spatial domain identification and tissue organization analysis.

More recently, several methods have been developed to integrate spatial multi-omics data. SpatialGlue (Long *et al.* 2024), PRAGA (Huang *et al.* 2025), and MISO (Coleman *et al.* 2025) have marked a significant step forward. However, these advanced frameworks can be characterized by two key limitations. First, their integration strategies often rely on concatenation or post hoc mapping of pre-computed, modality-specific views. This approach tends to capture only superficial correspondences rather than modeling intrinsic biological relationships, thus failing to establish a fully shared semantic space. Second, cross-modal alignment and local structural regularization are often handled separately rather than jointly within a unified objective. This separation can leave the final embeddings fragmented and susceptible to noise, potentially obscuring biologically coherent patterns across spatial regions. These limitations underscore the critical need for a new approach that can resolve both challenges simultaneously.

Here, we propose SpaAlign, a two-stage deep learning framework designed to identify biologically meaningful spatial domains through deep integration of spatial multi-omics data. In the first stage, SpaAlign constructs independent autoencoders for each modality and aligns cross-modal representations through a multi-objective pretraining strategy. Inspired by CLIP (Contrastive Language-Image Pretraining) (Radford *et al.* 2021), this strategy uses a cross-modal contrastive loss to embed features into a unified semantic space, while reconstruction and intra-modality consistency losses preserve modality-specific information and local structure. Following pretraining, we explicitly model cross-modal interactions by computing the outer product of the embeddings. In the second stage, SpaAlign introduces a deep clustering module that jointly refines the trainable components by adding a Kullback-Leibler (KL) divergence-based clustering loss. This joint optimization strategy further refines the representations, guiding the embeddings toward a more distinct cluster structure. To validate the effectiveness of SpaAlign, we conducted extensive experiments on simulated datasets as well as real-world spatial multi-omics datasets from human lymph node, mouse brain, and human tonsil. Comparisons with multiple existing methods demonstrate the robust and accurate performance of SpaAlign in spatial domain identification.

## 2 Results

### 2.1 Overview of SpaAlign

**Figure 1:**
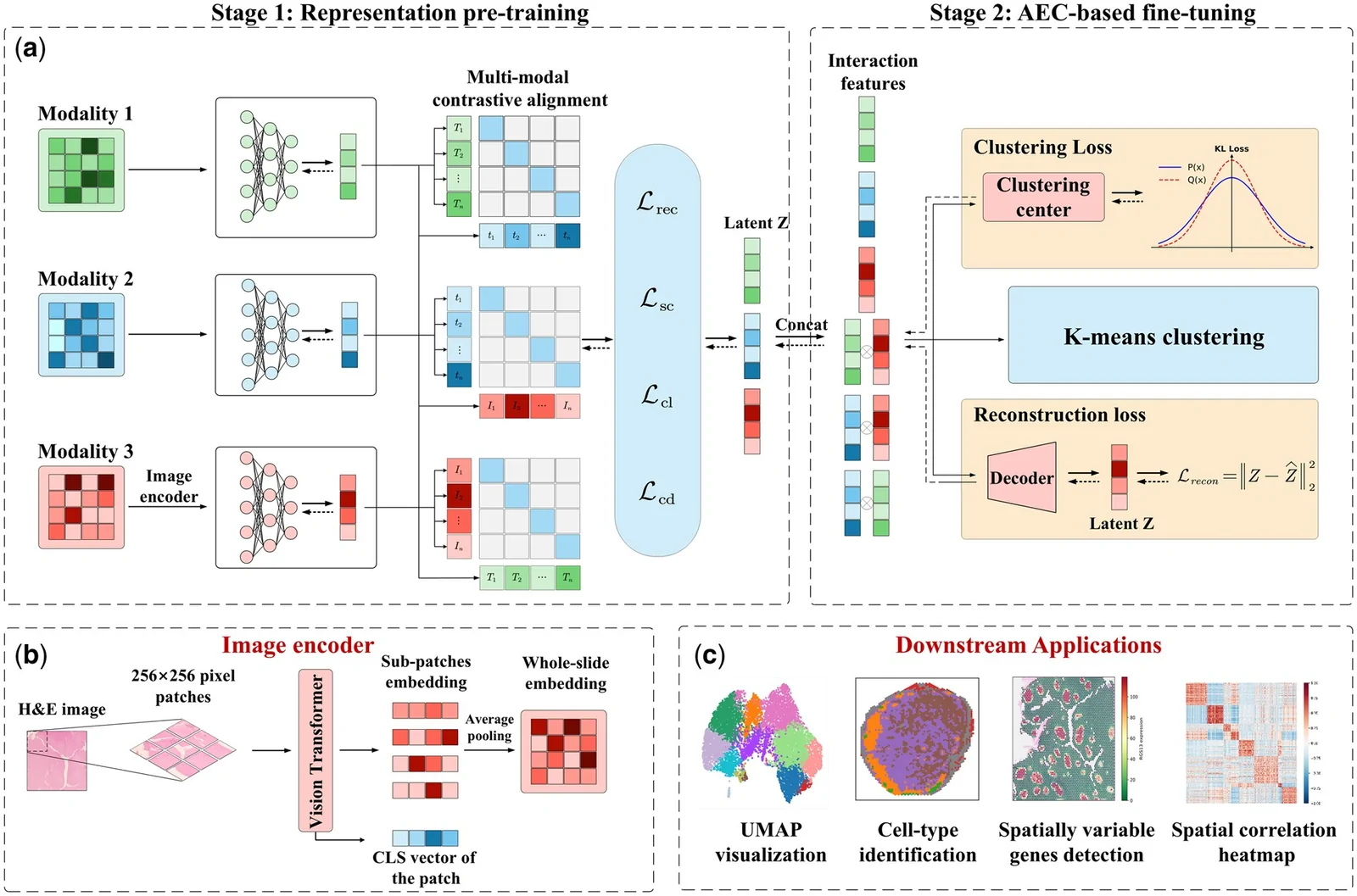
Overview of the SpaAlign framework. (a) The two-stage training pipeline. In Stage 1 (representation pretraining), modality-specific encoders generate latent representations (*Z*) and are optimized using a reconstruction loss ($L_{\text{rec}}$), an intra-modality consistency loss ($L_{\text{sc}}$), and a cross-modal contrastive alignment loss ($L_{\text{CLIP}}$), where $L_{\text{CLIP}}=\lambda_{1}L_{\text{cl}}+\lambda_{2}L_{\text{cd}}$. Here, $L_{\text{cl}}$ is a symmetric InfoNCE loss that enforces global semantic alignment between modalities, and $L_{\text{cd}}$ is a margin-based distance regularization loss. In Stage 2 (fine-tuning), PCA-reduced interaction features derived from pairwise tensor outer products of *Z* are concatenated with the latent representations. The trainable components are then jointly refined using the pretraining objective and a KL divergence-based clustering loss. (b) Architecture of the hierarchical image encoder. An H&E image is partitioned into 256×256 pixel patches. A Vision Transformer then processes each patch to extract multi-level features to capture both local and global context. (c) Downstream applications of the final embeddings, including UMAP visualization, cell-type identification, spatially variable gene (SVG) detection, and spatial correlation analysis.

SpaAlign is designed to identify biologically meaningful spatial domains by generating deeply integrated representations from spatial multi-omics and histological data (Fig. 1a). At its core, SpaAlign adopts a two-stage analytical framework. For histological inputs, a Vision Transformer (ViT) extracts hierarchical features from 256×256 pixel patches to capture key morphological contexts (Fig. 1b). In the first stage, cross-modal representations are aligned through multi-objective pretraining. In the second stage, these embeddings are jointly optimized and refined via deep clustering.

During the first stage, SpaAlign constructs a separate autoencoder for each modality. These are trained using reconstruction and intra-modality consistency terms to preserve modality-specific information and local structure, and a CLIP-inspired cross-modal contrastive loss to achieve precise alignment. To capture synergistic relationships across modalities, SpaAlign computes pairwise outer products of the pretrained embeddings to form a composite representation.

In the second stage, a deep clustering module jointly fine-tunes the trainable network components. By integrating a Kullback-Leibler (KL) divergence-based clustering loss, this joint optimization leverages the model’s own high-confidence predictions as supervisory signals. This promotes coherence and guides the embeddings toward a more distinct cluster structure.

The final output is a refined latent embedding that integrates all modalities while faithfully preserving spatial and functional structures. These robust embeddings serve as a versatile foundation for various downstream analyses, including UMAP visualization, cell-type identification, domain-enriched gene analysis, and spatial correlation analysis (Fig. 1c).

### 2.2 Assessing SpaAlign’s integration performance and robustness on simulated data

**Figure 2:**
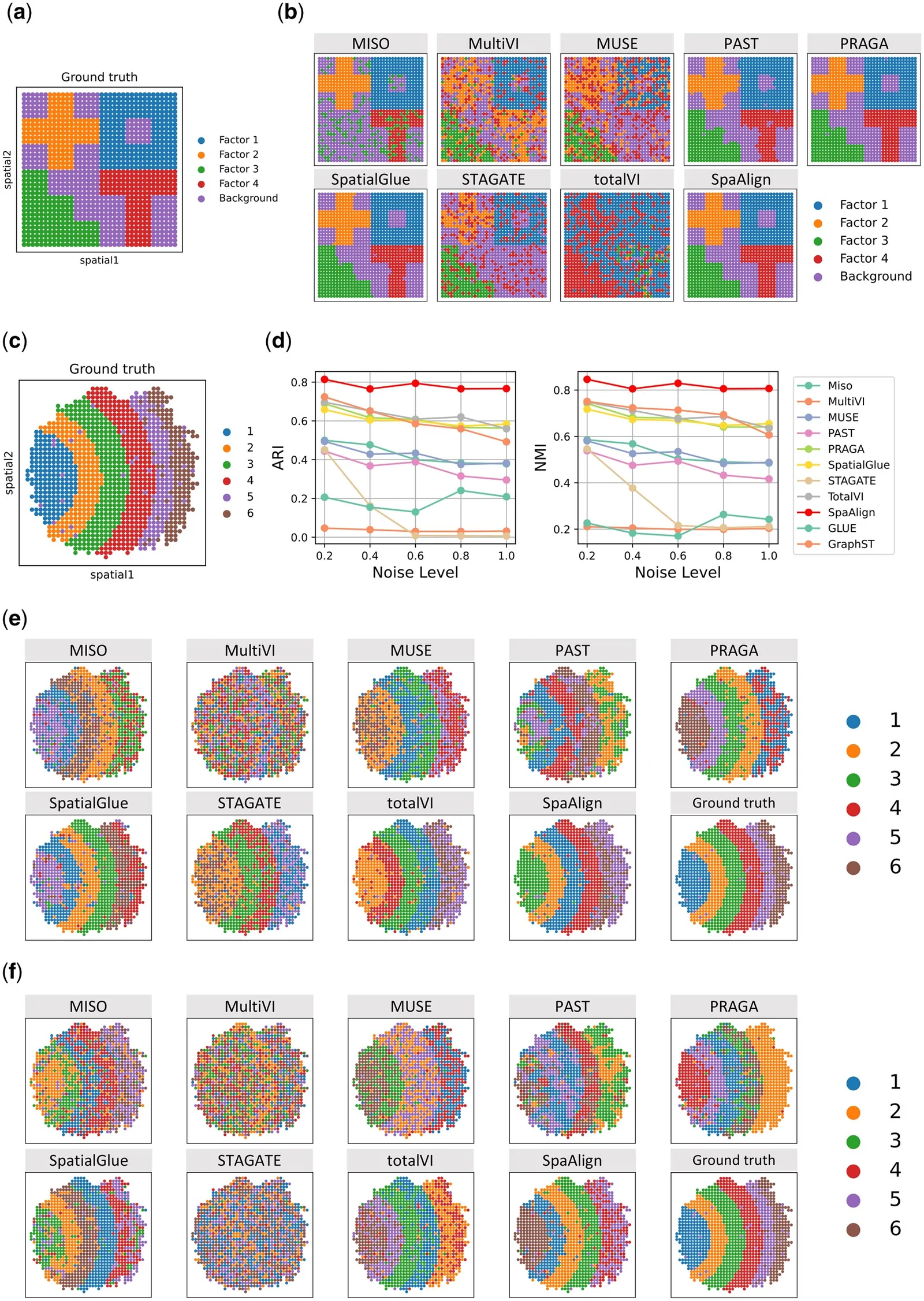
Evaluation of SpaAlign’s robustness to noise for spatial domain identification in simulated datasets. (a) Annotation of the simulated dataset. (b) Clustering results of the simulated data obtained by single-cell and spatial multi-omics methods (MISO, MultiVI, MUSE, PAST, PRAGA, SpatialGlue, STAGATE, totalVI, and SpaAlign). (c) Spatial domain annotation of another simulated dataset. (d) Quantitative evaluation under increasing noise levels, measured by ARI (left) and NMI (right). (e) Clustering results under intrinsic noise level = 0.2. (f) Clustering results under intrinsic noise level = 1.0.

We first benchmarked SpaAlign alongside competing spatial multi-omics integration methods using simulated data with known ground truth labels. The simulated dataset used in this benchmark, adopted from SpatialGlue (Long *et al.* 2024), comprises two modalities designed to simulate transcriptome and proteome layers, respectively. Modality 1 follows a zero-inflated negative binomial (ZINB) distribution and encodes spatial factors 1, 3, and 4, while modality 2 follows a negative binomial (NB) distribution and is uniquely informative for factor 2 (Fig. 2a), mimicking scenarios where different omics layers capture complementary yet non-redundant biological signals. Successful recovery in this scenario thus requires effective cross-modal integration. Performance was evaluated with a suite of supervised clustering metrics, including Mutual Information (MI) (Strehl and Ghosh 2002), Normalized Mutual Information (NMI) (Estévez *et al.* 2009), Adjusted Mutual Information (AMI) (Vinh *et al.* 2010), Fowlkes-Mallows Index (FMI) (Fowlkes and Mallows 1983), Adjusted Rand Index (ARI) (Steinley 2004), V-Measure (Rosenberg and Hirschberg 2007), and Completeness (Rosenberg and Hirschberg 2007). The baseline methods included in this comparison were MISO (Coleman *et al.* 2025), MultiVI (Ashuach *et al.* 2023), PAST (Li *et al.* 2023), MUSE (Bao *et al.* 2022), PRAGA (Huang *et al.* 2025), SpatialGlue (Long *et al.* 2024), STAGATE (Dong and Zhang 2022), GraphST (Long *et al.* 2023), GLUE (Cao and Gao 2022) and totalVI (Gayoso *et al.* 2021), each applied using its standard configuration.

Qualitatively, SpaAlign recovered all four spatial factors with boundaries closely aligned to the ground truth (Fig. 2b; Fig. S1). In contrast, competing methods showed varying degrees of fragmentation or boundary ambiguity. This qualitative superiority was supported by quantitative analysis, which positioned SpaAlign as the top-performing method (Fig. S2). SpaAlign uniquely achieved the highest scores across NMI, AMI, and ARI, accurately recovering the ground-truth labels.

To further assess robustness, we generated another simulated dataset using the scMultiSim R package (Li *et al.* 2025), which simulates multi-omics single-cell datasets containing RNA and ATAC modalities based on gene regulatory networks (GRNs) and differentiation trees. The dataset comprised six cell clusters with distinct transcriptomic and epigenomic profiles (Fig. 2c). To evaluate robustness under different noise conditions, we varied the intrinsic noise parameter from 0.2 to 1.0, which controls the level of intrinsic transcriptional randomness during count generation. Higher intrinsic noise increases stochastic variation in gene expression and makes cross-modal alignment and domain identification more challenging.

Across all noise levels, SpaAlign consistently outperformed all baseline methods, while maintaining strong robustness, with its ARI showing an absolute decrease of only 4.8% as the noise level increased. In comparison, GraphST showed a substantial absolute decrease of 23.2% in ARI from its initial performance, while GLUE consistently underperformed across all settings. Notably, STAGATE collapsed under high-noise conditions, exhibiting a dramatic decline in ARI. Qualitatively, SpaAlign preserved high structural coherence consistent with the ground truth at both noise extremes (Fig. 2e and 2f; Fig. S3), avoiding the severe fragmentation observed in baseline outputs. UMAP visualizations further confirmed SpaAlign’s uniquely well-separated embeddings under all conditions (Figs. S4 and S5).

### 2.3 SpaAlign delineates complex and multi-layered tissue architectures on the human lymph node dataset

**Figure 3:**
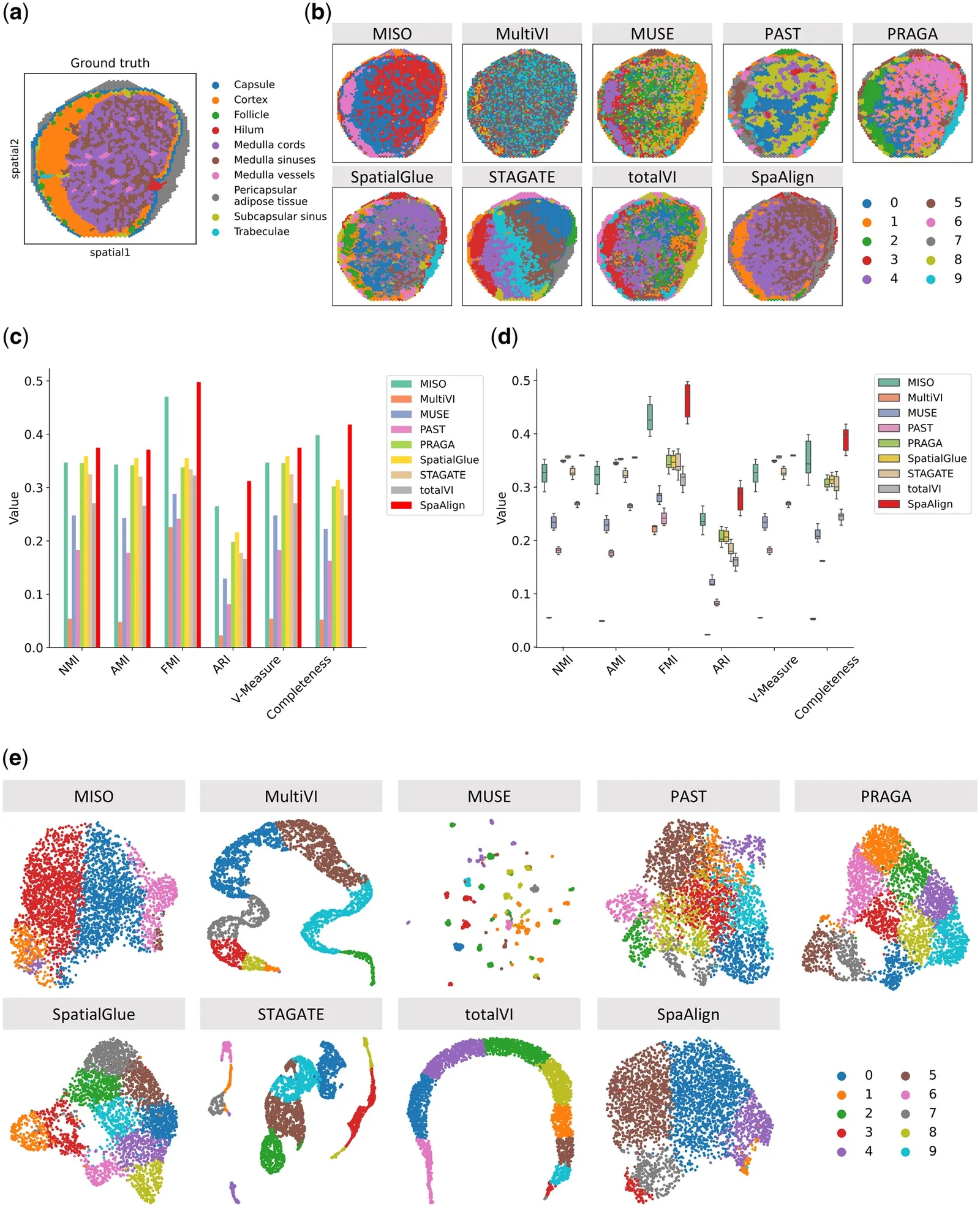
Performance of SpaAlign on the human lymph node spatial multi-omics dataset. (a) Ground-truth histological annotation of major lymph node structures. (b) Clustering outputs from all methods. (c) Comparison of clustering accuracy with the number of clusters fixed to the annotated value (n=10). (d) Quantitative evaluation across different clustering resolutions (8–12 clusters). (e) UMAP visualization of learned embeddings from all methods.

We benchmarked SpaAlign on a 10× Visium human lymph node dataset comprising spatially matched RNA and protein measurements. This dataset includes H&E-based manual annotations of major anatomical structures—such as the cortex, medulla, capsule, and follicles—providing a robust ground truth for spatial domain recovery (Fig. 3a).

SpaAlign exhibited the highest anatomical fidelity, uniquely recapitulating the fundamental capsule-cortex-medulla architecture and fine-scale domains like follicles and pericapsular adipose tissue (Fig. 3b; Fig. S6). In contrast, baselines exhibited significant limitations: MultiVI and totalVI lacked coherent spatial organization, MUSE and STAGATE produced fragmented or merged segmentations, and PAST and PRAGA displayed boundary ambiguities and missed internal details.

Quantitatively, at the reference resolution (n=10), SpaAlign consistently outperformed all baselines across six metrics (Fig. 3c; Fig. S6). In particular, SpaAlign achieved the highest ARI, outperforming MISO, GraphST, and GLUE by absolute margins of 4.7%, 8.7%, and 15.6%, respectively. SpaAlign also demonstrated superior stability with low variance across varying clustering resolutions (n=8−12, Fig. 3d). Furthermore, UMAP visualizations (Fig. 3e) confirmed that SpaAlign produced compact, well-separated clusters, whereas baselines generated diffuse, elongated (STAGATE, totalVI), or overlapping (MISO, SpatialGlue) embeddings.

An ablation study (Fig. S7) confirmed the indispensable roles of SpaAlign’s core components. Removing the CLIP-based contrastive alignment module alone caused an absolute ARI decrease of 4.8%. Jointly removing both the CLIP and AEC-based deep clustering modules caused an absolute ARI decrease of 7.8% and notable declines across all other metrics. These findings validate their complementary functions in achieving superior embedding quality.

Furthermore, a comprehensive hyperparameter sensitivity analysis on the Human Lymph Node dataset (Fig. S8) demonstrated that SpaAlign’s performance remains stable across a wide range of settings for key hyperparameters (α, γ, σ, and tol), confirming the robustness of the proposed framework.

### 2.4 SpaAlign integrates sparse epigenome-transcriptome data to resolve fine-grained structures

**Figure 4:**
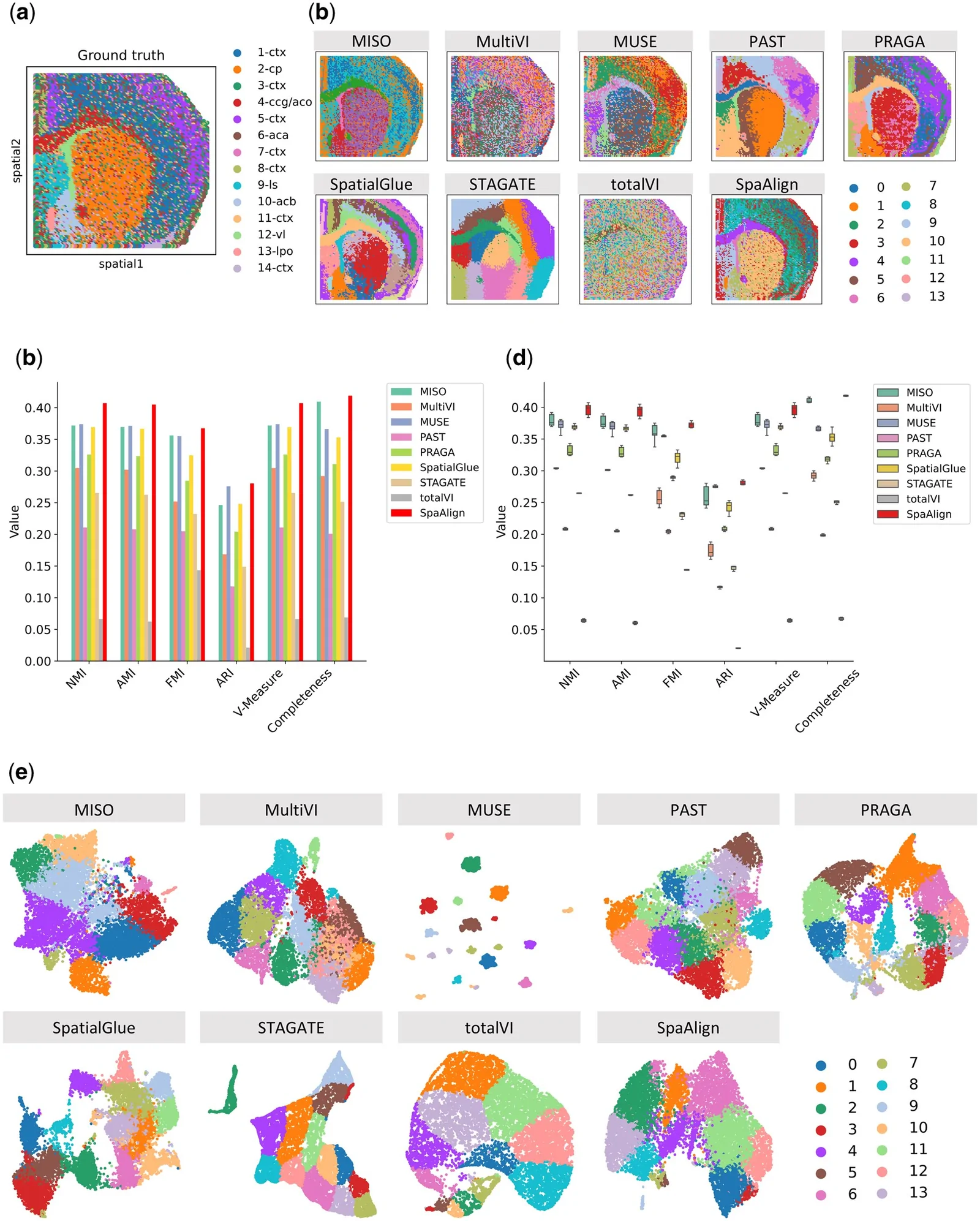
Performance comparison of SpaAlign and other methods for delineating anatomical domains in mouse brain data. (a) Annotation reference of mouse brain coronal sections from the Allen Mouse Brain Atlas. (b) Visualization of spatial domain prediction across unimodal, single-cell, and spatial multi-omics integration methods. (c) Comparison of clustering accuracy with the number of clusters fixed to the annotated value (n=14). (d) Box plots of six supervised metrics, including clustering result scores, with the number of clusters ranging from 12 to 16. (e) UMAP visualization of learned embeddings showing spatial domain prediction for all evaluated methods, with the number of domains fixed at the annotated value of 14.

We next evaluated SpaAlign on a spatial ATAC-RNA dataset from a P22 mouse brain coronal section, a challenging testbed due to high anatomical complexity and chromatin data sparsity. Using the Allen Mouse Brain Atlas as a reference, we assessed its ability to resolve fine-grained anatomical structures (e.g. cortex, caudate putamen, lateral ventricle) (Fig. 4a).

As shown in Figs. 4b and S9, SpaAlign successfully reconstructed biologically coherent brain regions, uniquely resolving both cortical layers and fine subcortical structures with high fidelity. Conversely, baselines suffered from severe fragmentation (MUSE, totalVI, STAGATE) or indistinct boundaries (SpatialGlue, PRAGA). This superior organization seamlessly extended to the latent space: UMAP visualizations (Fig. 4e) confirmed that SpaAlign produced compact, anatomically aligned clusters, contrasting sharply with the diffuse (MUSE) or severely entangled (STAGATE, totalVI) embeddings of competing methods.

Quantitatively, at the reference resolution (n=14), SpaAlign achieves the highest NMI and ARI (Fig. 4c; Fig. S9). For NMI, SpaAlign achieved an absolute improvement of 3.0% over the second-best method, MUSE. Furthermore, SpaAlign maintained top-tier stability with low variance across varying clustering resolutions (n=12−16, Fig. 4d), whereas competing methods degraded substantially at non-optimal settings.

### 2.5 SpaAlign identifies biological microstructures through tri-modal data integration

**Figure 5:**
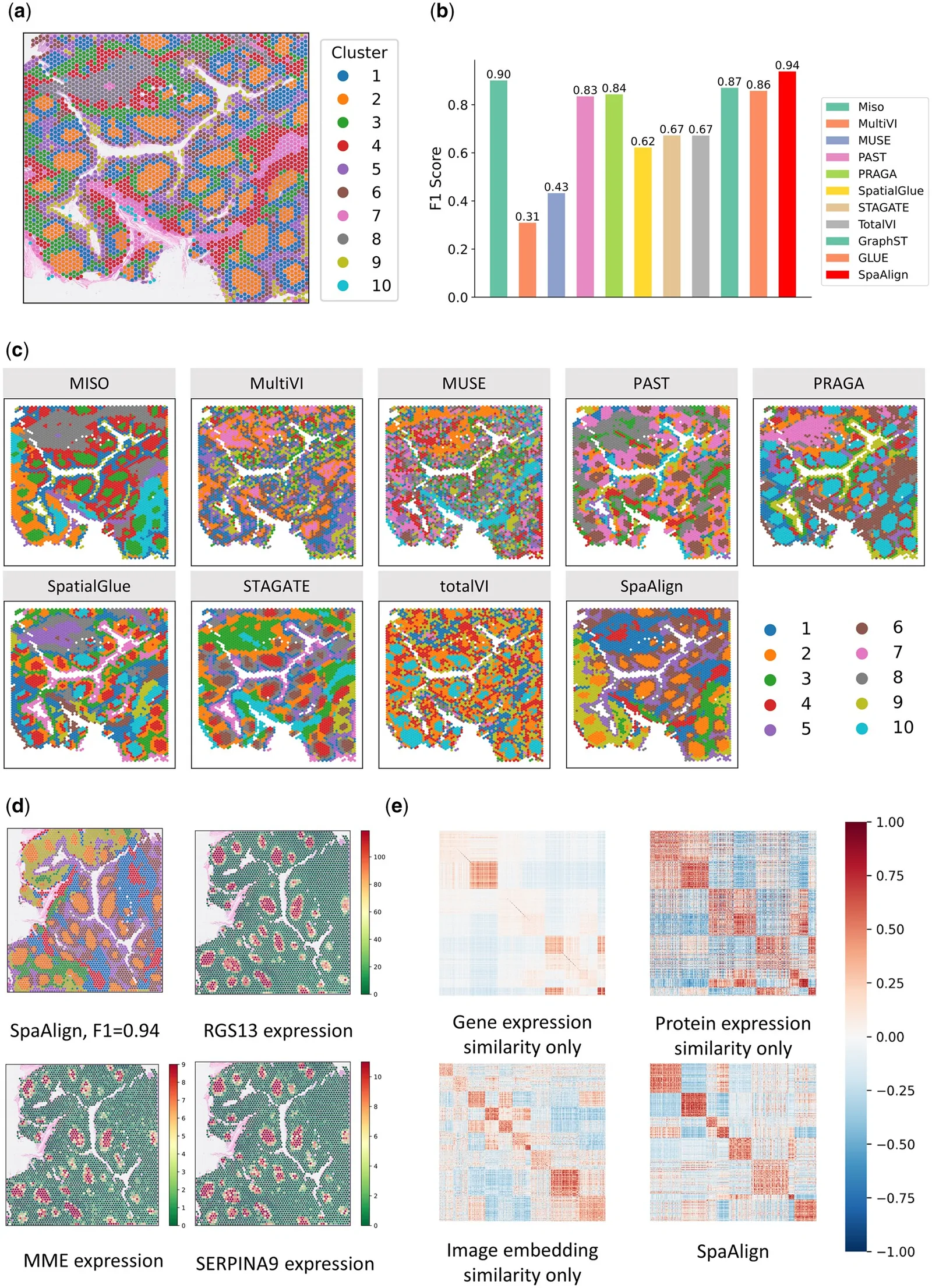
Performance on human tonsil spatial transcriptomics, protein, and histology data. (a) Manual histological annotation of the human tonsil on the H&E-stained image, delineating ten spatial domains. (b) Quantitative comparison of clustering accuracy in the germinal center (label 2), measured by F1 score. (c) Clustering outputs from baseline methods (MISO, MultiVI, MUSE, PAST, PRAGA, SpatialGlue, STAGATE, and totalVI) compared with SpaAlign. SpaAlign, MISO, and MUSE use three modalities (RNA, histology, and protein); the other methods use two modalities (RNA and protein). (d) SpaAlign clustering results (F1 = 0.94) alongside representative marker gene expression patterns (RGS13, MME, SERPINA9), confirming biological consistency of the recovered germinal center region. (e) Similarity heatmaps constructed from single-modality features (gene expression only, protein expression only, image embeddings only) versus SpaAlign’s multi-omics integration.

To evaluate tri-modal integration for identifying tissue microstructures, we applied SpaAlign to a human tonsil dataset comprising spatial transcriptomics, proteomics, and H&E histology. With germinal centers well-annotated by histology and molecular markers, this dataset serves as a rigorous benchmark for spatial localization (Fig. 5a).

We benchmarked SpaAlign against ten baseline methods. SpaAlign, MISO, and MUSE utilized all three modalities, while the remaining methods were restricted to RNA and protein because they lack architectural support for direct histological image integration. Notably, despite their general competitiveness, the inability of PRAGA and SpatialGlue to incorporate image-derived structural cues limits their precision in fine-grained tasks like germinal center delineation.

SpaAlign achieved the most accurate germinal center (GC) delineation with coherent boundaries and minimal spillover, whereas MUSE, STAGATE, and totalVI produced highly fragmented or overlapping clusters (Fig. 5c; Fig. S10). Using an F1-based metric for GC identification (Fig. 5b), SpaAlign achieved the highest score, outperforming MISO, the second-best method. Notably, bi-modal methods performed moderately, while approaches excluding histology showed significantly weaker correspondence. These results underscore the advantage of SpaAlign’s contrastive alignment and interaction-based design in fusing tri-modal data for fine-grained spatial domain identification.

Analysis of domain-enriched genes further validated the biological significance of SpaAlign’s identified spatial domains (Fig. 5d). Differential expression analysis was performed on the original gene expression matrix using the Wilcoxon rank-sum test to identify domain-specific marker genes. The identified GC regions showed strong enrichment for known GC markers such as RGS13 and MME, while SERPINA9 exhibited a distinct complementary pattern in surrounding mantle zone domains. These biologically meaningful spatial expression patterns demonstrate that SpaAlign accurately recovers transcriptionally distinct microenvironments.

To further investigate the structural organization of the learned representations, we visualized the latent space using UMAP and spot-by-spot Pearson correlation heatmaps computed from the learned embeddings (Fig. 5e, Fig. S11). While single-modality embeddings appeared diffuse and lacked clear structural separation, SpaAlign’s integrated representation exhibited a pronounced block-diagonal correlation structure, indicating strong within-domain similarity and clear between-domain separation. These results suggest that multi-modal integration facilitates the recovery of more coherent and spatially organized representations, thereby supporting more accurate spatial domain identification.

To validate SpaAlign’s architectural design, we conducted ablation experiments on the tonsil dataset. Without histological data, SpaAlign already outperformed all competing methods, achieving the highest F1 score for germinal center identification. Incorporating histological data further improved performance, resulting in an absolute F1 improvement of 3.0%, primarily driven by enhanced recall while maintaining high precision. Furthermore, we benchmarked four state-of-the-art vision models [HIPT (Chen *et al.* 2022), omiCLIP (Chen *et al.* 2025), Prov-GigaPath (Xu *et al.* 2024), and UNI (Chen *et al.* 2024)] as feature encoders (Fig. S12). HIPT achieved the highest F1 score, outperforming the second-best vision encoder, while providing the optimal balance between precision and recall. In contrast, UNI suffered from substantially lower recall despite maintaining high precision. Consequently, HIPT was selected as the default image encoder for all subsequent analyses.

To quantify the distinct contribution of the protein modality to spatial-domain clustering, we conducted a systematic ablation study on the human tonsil dataset by evaluating performance across different modality combinations. Compared with RNA-only analysis, integrating surface protein measurements yielded absolute improvements of 2.3% and 9.6% in ARI and F1 score, respectively, with similarly consistent improvements observed across NMI, AMI, FMI, V-measure, and Completeness. These results highlight the complementary role of proteomic information in alleviating the intrinsic sparsity of transcriptomic profiles. Furthermore, jointly integrating transcriptomic, proteomic, and spatial imaging information achieved the best overall performance (Fig. S13), yielding absolute improvements of 5.8% and 15.8% in ARI and F1 score, respectively, compared with RNA-only analysis. These findings demonstrate that synergistic multi-modal integration is critical for accurately delineating heterogeneous tissue microenvironments.

In addition, we extended the evaluation to another tri-modal spatial multi-omics dataset, the HumanBreast dataset, which also comprises spatial transcriptomics, proteomics, and H&E histology (Zhao *et al.* 2021). SpaAlign produced biologically coherent spatial domains with well-separated boundaries (Fig. S14a). Quantitatively, SpaAlign achieved the best performance across all six supervised clustering metrics, outperforming the second-best method, PAST, by an absolute ARI margin of 11.9% (Fig. S14b). These results confirm that SpaAlign’s tri-modal integration advantage is robust across diverse tissue types.

## 3 Discussion

SpaAlign addresses the challenge of spatial multi-omics integration by decoupling semantic alignment from structural refinement. Unlike traditional linear concatenation or *post hoc* mapping, our CLIP-inspired contrastive stage establishes a shared semantic space where heterogeneous modalities—including transcriptomics, proteomics, epigenomics, and histology—become directly comparable. This is synergistically coupled with a self-supervised clustering objective that enforces spot-level coherence. By leveraging high-confidence predictions to refine embeddings, SpaAlign effectively mitigates noise and reveals latent biological patterns, providing a robust theoretical foundation for high-fidelity spatial domain identification.

While advanced methods like SpatialGlue, MISO, and PRAGA effectively align heterogeneous modalities, they often treat each as an independent “view,” potentially hindering the formation of a fully shared semantic space. Moreover, cross-modal semantic alignment and local structural regularization may not be jointly coupled within a unified objective. SpaAlign combines these objectives to support both cross-modal comparability and locally coherent representations.

SpaAlign’s efficacy was validated across diverse tissue types and modalities. It demonstrated superior structural precision by recapitulating the capsule-cortex-medulla architecture in lymph nodes and achieving top-tier germinal center identification in tri-modal tonsil data. Moreover, its high-fidelity resolution of fine-grained structures in sparse mouse brain epigenome-transcriptome data underscores the framework’s robustness and scalability for complex spatial multi-omics integration.

Regarding computational scalability, the modality-specific encoders and affinity-based regularizers scale linearly with the number of modalities *M*, as each modality is processed independently. In contrast, the cross-modal contrastive alignment and interaction fusion require pairwise operations across all modality pairs, yielding $\left(\begin{array}{c} M\\ 2 \end{array}\right)$ contrastive comparisons and outer-product computations, which results in an overall complexity of $O(M^{2})$ with respect to modality count. For instance, extending from three to four modalities increases the number of contrastive pairs from three to six and doubles the fused feature dimension. This quadratic cost could be mitigated in future work through hierarchical fusion strategies that cluster similar modalities prior to cross-modal alignment.

Beyond domain identification, SpaAlign generates a high-quality, unified latent embedding that serves as a versatile foundation for downstream analyses. By capturing both molecular and morphological information in a coherent representation, it enables precise cell-type annotation, detection of functional SVGs, and construction of cross-modal spatial correlation maps, thereby facilitating a deeper exploration of the tissue microenvironment.

Furthermore, the shared latent representation and modality-specific decoding architecture of SpaAlign provide a natural foundation for future extensions toward cross-omics inference and reference-guided spatial modeling. In principle, the learned cross-modal semantic space could be coupled with conditional or directional decoding strategies to enable tasks such as modality completion or prediction of proteomic and epigenomic profiles from spatial transcriptomic representations (Cao *et al.* 2024, Lang *et al.* 2026). In addition, incorporating prior knowledge from single-cell transcriptomic references or large-scale cell atlases (Shengquan *et al.* 2021, Li *et al.* 2023) may further alleviate data sparsity, improve spatial resolution, and enhance the robustness and biological interpretability of spatial multi-omics integration. Such extensions could broaden the applicability of SpaAlign to downstream analyses including modality completion, cross-modal prediction, spatial signal imputation, and refined cell-type or spatial-domain characterization.

Despite its performance, SpaAlign has several limitations that offer avenues for future work. Currently purely data-driven, the framework could benefit from integrating biological prior knowledge, such as gene regulatory networks, to enhance interpretability. Additionally, leveraging single-cell foundation models [e.g. scFoundation (Hao *et al.* 2024), CellFM (Zeng *et al.* 2025)] and implementing mechanisms to automatically weight modality contributions or down-weight low-quality data would further refine its integrative capabilities and impact.

## 4 Methods

SpaAlign comprises six architectural components: modality-specific affinity graphs, autoencoders, consistency regularization, cross-modal contrastive alignment, interaction fusion, and adaptive embedding clustering. These are trained in two stages: Stage 1 optimizes representation learning and cross-modal alignment; Stage 2 jointly refines the trainable components. Detailed specifications are summarized in Appendix Tables S1 and S2.

### 4.1 Data preprocessing and graph construction

SpaAlign applies modality-specific preprocessing. RNA and ATAC matrices are filtered and log-transformed, whereas protein matrices undergo protein-specific log normalization. Each input is subsequently *Z*-score normalized. H&E-stained images: We use the Hierarchical Image Pyramid Transformer (HIPT) (Chen *et al.* 2022) to extract morphological features. Local 256×256 pixel patches are encoded into 576-dimensional tokens and aggregated into spot-level embeddings through a global context-aware ViT and average pooling.

Graph Construction: For each modality *m*, we construct an affinity graph from its standardized features. Given feature vectors $x_{i}^{\left(m\right)}$ and $x_{j}^{\left(m\right)}$, their affinity is:


(1) $$A_{ij}^{\left(m\right)}=\text{exp}\;\left(-\frac{\|x_{i}^{\left(m\right)}-x_{j}^{\left(m\right)}{\|}^{2}}{2\sigma^{2}}\right)$$

where σ controls affinity decay and is 30 by default. The full-pairwise formulation is used by default. For large datasets, the graph can be sparsified by retaining the *k* nearest neighbors in the modality feature space (k=100 by default):


(2) $$A_{ij}^{\left(m\right)}=\{\begin{array}{cc} \;\text{exp}\;\left(-\frac{\|x_{i}^{\left(m\right)}-x_{j}^{\left(m\right)}{\|}^{2}}{2\sigma^{2}}\right), & j\in \text{KNN}(i),\\ 0, & \text{otherwise}. \end{array}$$

### 4.2 Modality-specific feature encoding

The primary objective of the encoding stage is the transformation of heterogeneous modality-specific information into a machine-interpretable, low-dimensional format. SpaAlign employs independent encoders for each modality to account for their distinct feature spaces and dynamic ranges. When the feature dimension exceeds 128, $X^{\left(m\right)}$ is projected into a 128-dimensional subspace by PCA; otherwise, the normalized input is retained. A two-layer linear encoder then produces the latent representation:


(3) $$h_{i}^{\left(m\right)}=W_{1}^{\left(m\right)}x_{i}^{\left(m\right)}+b_{1}^{\left(m\right)}$$

(4) $$z_{i}^{\left(m\right)}=W_{2}^{\left(m\right)}h_{i}^{\left(m\right)}+b_{2}^{\left(m\right)}$$

where $z_{i}^{\left(m\right)}\in R^{32}$. A linear decoder reconstructs the input as ${\hat{x}}_{i}^{\left(m\right)}=W_{d}^{\left(m\right)}z_{i}^{\left(m\right)}+b_{d}^{\left(m\right)}$, with $L_{\text{rec}}^{\left(m\right)}=N^{-1}\sum_{i}\|x_{i}^{\left(m\right)}-{\hat{x}}_{i}^{\left(m\right)}{\|}_{2}^{2}$. Orthogonal parametrization of the 32×32 matrix $W_{2}^{\left(m\right)}$, satisfying $W_{2}^{(m)⊤}W_{2}^{\left(m\right)}=I$, helps maintain distinct latent dimensions during alignment.

### 4.3 Intra-modality structural consistency

We use the affinity graph to regularize each latent manifold. The consistency loss penalizes latent discrepancies between highly similar spots:


(5) $$L_{\text{sc}}^{\left(m\right)}=\frac{1}{C_{m}}\sum_{(i,j)\in E_{m}}A_{ij}^{\left(m\right)}\|z_{i}^{\left(m\right)}-z_{j}^{\left(m\right)}{\|}_{2}$$

where $E_{m}$ contains upper-triangular pairs in the dense setting and retained edges in the sparse setting; $C_{m}$ is the normalization factor, set to $N^{2}$ for dense matrices and $\left|E_{m}\right|$ for sparse matrices, respectively. This term suppresses localized noise and promotes locally coherent embeddings.

### 4.4 Cross-modal contrastive alignment

The integration core lies in aligning the latent spaces derived from different modalities into a shared semantic framework. SpaAlign bridges the modality gap through a dual-objective contrastive framework that maximizes the mutual information between paired molecular and morphological signals. We optimize a joint alignment loss $L_{\text{CLIP}}=\lambda_{1}L_{\text{cl}}+\lambda_{2}L_{\text{cd}}$, which balances global semantic consistency with local geometric precision. For an ordered modality pair, the directional InfoNCE term is:


(6) $$\ell_{\text{cl}}^{\left(m_{1}\to m_{2}\right)}=-\frac{1}{B}\sum_{i=1}^{B}\;\text{log}\;\frac{\;\text{exp}(\text{sim}(z_{i}^{\left(m_{1}\right)},z_{i}^{\left(m_{2}\right)})/\tau )}{\sum_{j=1}^{B}\;\text{exp}\;(\text{sim}(z_{i}^{\left(m_{1}\right)},z_{j}^{\left(m_{2}\right)})/\tau )}$$

The symmetric pair loss is $L_{\text{cl}}^{\left(m_{1},m_{2}\right)}=[\ell_{\text{cl}}^{\left(m_{1}\to m_{2}\right)}+\ell_{\text{cl}}^{\left(m_{2}\to m_{1}\right)}]/2$, and $L_{\text{cl}}$ sums it over all ordered modality pairs. The temperature τ is 1.0. Complementing this, we propose a margin-based distance regularization $L_{\text{cd}}$ to address the limitations of cosine similarity, which only considers angular correlation. By explicitly constraining Euclidean distances:


(7) $$\ell_{\text{cd}}^{\left(m_{1},m_{2}\right)}=\frac{1}{B}\sum_{i=1}^{B}D_{ii}+\frac{1}{B(B-1)}\sum_{i\neq j}\text{max}(0,\delta -D_{ij})$$

With δ=1.0, this term pulls corresponding spots together and separates off-diagonal pairs; its global separation effect is balanced by $L_{\text{sc}}$, which preserves local relationships. $L_{\text{cd}}$ is summed over all unordered modality pairs.

### 4.5 Cross-modal interaction fusion

For every pair in $C=\{(m_{1},m_{2}):1\leq m_{1}<m_{2}\leq M\}$, we compute an outer-product interaction tensor:


(8) $$I_{i}^{\left(m_{1},m_{2}\right)}=z_{i}^{\left(m_{1}\right)}⊗z_{i}^{\left(m_{2}\right)}\in R^{32\times 32}$$

Each 1024-dimensional interaction map is reduced to 32 dimensions by PCA and concatenated with all latent embeddings: $f_{i}=[{\left\{z_{i}^{\left(m\right)}\right\}}_{m=1}^{M},{\left\{\text{PCA}(\text{vec}(I_{i}^{\left(m_{1},m_{2}\right)}))\right\}}_{(m_{1},m_{2})\in C}]$. During fine-tuning, these PCA features are recomputed, while encoder gradients pass through the concatenated latent embeddings.

### 4.6 Clustering with AEC refinement

The final objective of SpaAlign is the translation of integrated features into biologically discrete spatial domains. Adaptive Embedding Clustering (AEC) uses a Student’s *t*-distribution with degrees-of-freedom parameter α to calculate soft assignments between $f_{i}$ and centroid $\mu_{k}$:


(9) $$q_{ik}=\frac{{\left(1+\|f_{i}-\mu_{k}{\|}^{2}/\alpha \right)}^{-(\alpha +1)/2}}{\sum_{k^{\prime }}\left(1+\right\|f_{i}-\mu_{k^{\prime }}{\|}^{2}/\alpha {)}^{-(\alpha +1)/2}}$$

where α=1.0 by default. The target is $p_{ik}=(q_{ik}^{2}/f_{k})/\sum_{k^{\prime }}\left(q_{ik^{\prime }}^{2}/f_{k^{\prime }}\right)$, where $f_{k}=\sum_{i}q_{ik}$. We minimize:


(10) $$L_{\text{cluster}}=\sum_{i}\sum_{k}p_{ik}\;\text{log}\;\frac{p_{ik}}{q_{ik}}$$

This target emphasizes high-confidence assignments and suppresses low-confidence noise, promoting compact clusters.

### 4.7 Optimization and training strategy

The stability and performance of SpaAlign are ensured by a two-stage training strategy.

In Stage 1 (multi-objective pretraining), the model learns modality-specific representations and cross-modal alignment by minimizing:


(11) $$L_{\text{pretrain}}=\frac{1}{2}\left[\sum_{m}\left(L_{\text{rec}}^{\left(m\right)}+\beta L_{\text{sc}}^{\left(m\right)}\right)+\lambda_{1}L_{\text{cl}}+\lambda_{2}L_{\text{cd}}\right]$$

where β=1.0, $\lambda_{1}=0.9$, $\lambda_{2}=0.1$. The model is trained using Adam with a learning rate of $10^{-3}$.

In Stage 2, cluster centroids are initialized via *k*-means and the trainable components are jointly optimized using:


(12) $$L_{\text{total}}=L_{\text{pretrain}}+\gamma L_{\text{cluster}}$$

where γ=0.5. Fine-tuning uses Adam with a learning rate of $10^{-4}$ and stops when the fraction of changed assignments between updates falls below $\text{tol}=10^{-4}$.

## Supplementary Material

btag629_Supplementary_Data

## Data Availability

All datasets used in this study were obtained from publicly available sources. The 10× Visium human lymph node dataset was obtained from the Gene Expression Omnibus (GEO; accession number GSE263617, https://www.ncbi.nlm.nih.gov/geo/query/acc.cgi? acc=GSE263617). The spatial epigenome-transcriptome mouse brain dataset was sourced from AtlasXplore: https://web.atlasxomics.com/visualization/Fan/. The human tonsil tri-modal dataset is publicly available from 10× Genomics (https://www.10xgenomics.com/resources/datasets/gene-protein-expression-library-of-human-tonsil-cytassist-ffpe-2-standard). The human breast cancer dataset is publicly available from 10× Genomics (https://www.10xgenomics.com/datasets/invasive-ductal-carcinoma-stained-with-fluorescent-cd-3-antibody-1-standard-1-2-0). Additionally, the human lymph node, mouse brain, and simulation datasets, including those used for noise-robustness assessment, along with the SpaAlign code, are publicly available on GitHub: https://github.com/VitaIntelli-CQU/SpaAlign/tree/master/data.

## Code availability

All code used in this study is available at https://github.com/VitaIntelli-CQU/SpaAlign. The version used in this manuscript is archived under the DOI: https://doi.org/10.5281/zenodo.20250508.
