# Supplementary material for: Unified representation learning for spatial multi-omics
Source: Bioinformatics. 2026 Aug 24;42(9):btag629. doi: 10.1093/bioinformatics/btag629 (PMC13544968; doi:10.1093/bioinformatics/btag629)
Supplement: Supplementary file 1 [file btag629_supplementary_data.pdf]

Appendix: Supplementary Materials for SpaAlign

Table S1: SpaAlign architecture components and their roles.

**Component**	**Loss Term**	**Stage**
Modality Encoder (Sec. 4.2)	$\mathcal{L}_{\text{recon}}$	1, 2
Graph-Laplacian Regularizer (Sec. 4.3)	$\mathcal{L}_{sc}$	1, 2
Contrastive Alignment (Sec. 4.4)	$\mathcal{L}_{CLIP}$	1, 2
Interaction Fusion (Sec. 4.5)	–	1, 2
AEC Clustering (Sec. 4.6)	$\mathcal{L}_{\text{cluster}}$	2

Table S2: Two-stage training strategy.

**Stage**	**Optimized For**	**Loss**
Stage 1	Representation and alignment	$\mathcal{L}_{\text{pretrain}}$
Stage 2	Clustering refinement	$\mathcal{L}_{\text{total}}$

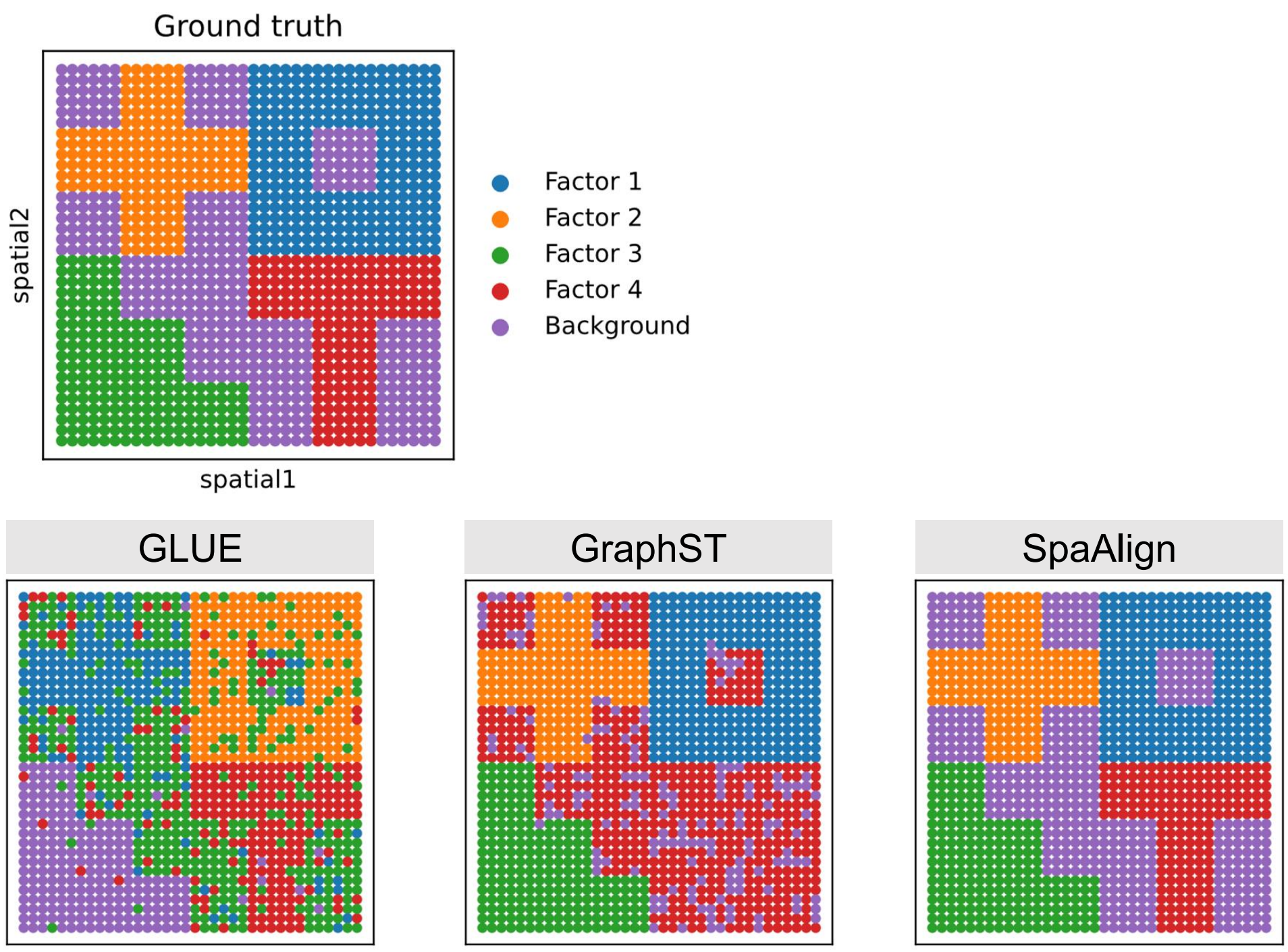

Fig. S1: **Ground-truth annotations and clustering results of the simulated dataset obtained by GLUE, GraphST, and SpaAlign.**

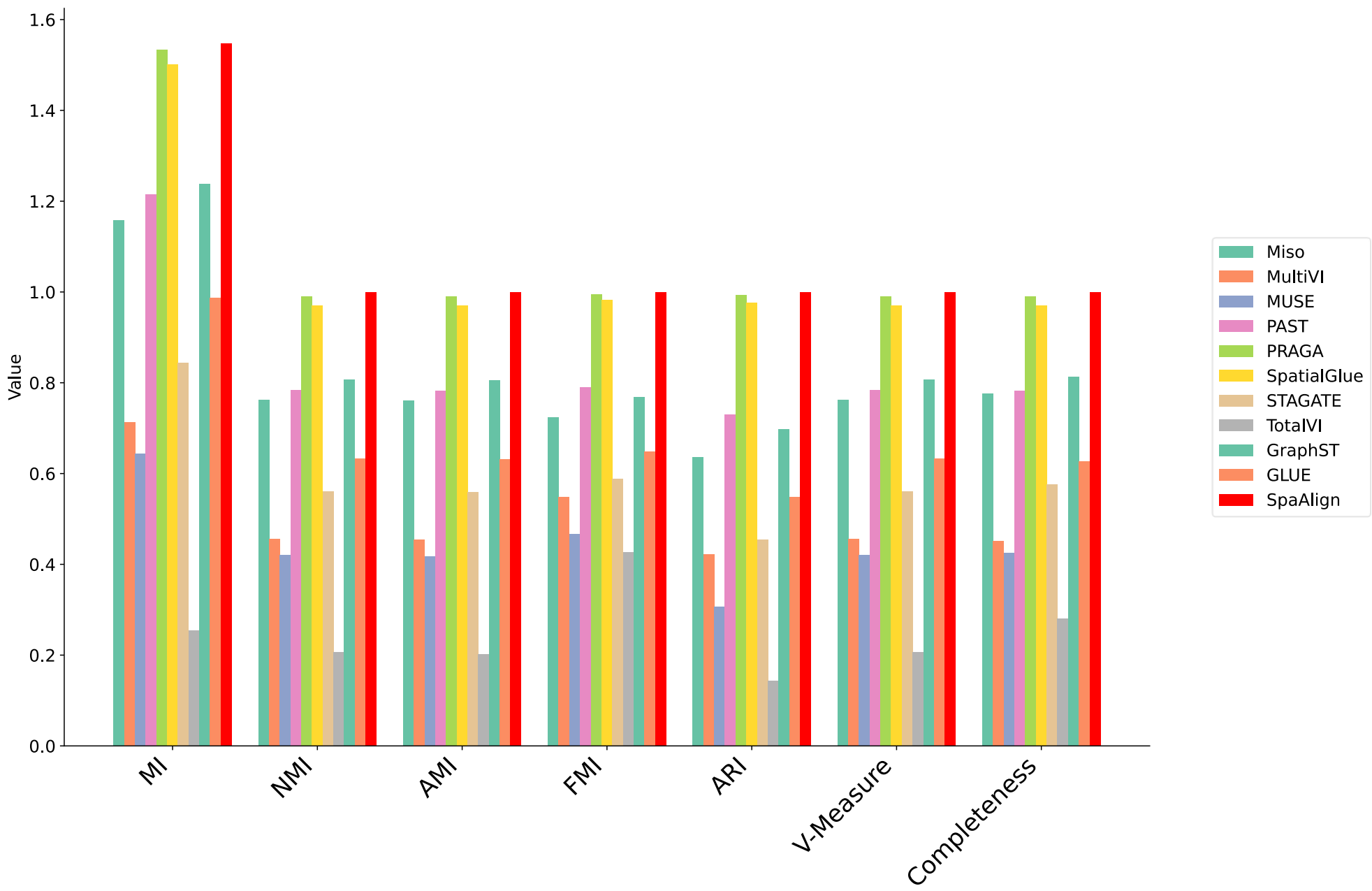

Fig. S2: **Quantitative benchmarking of SpaAlign against baseline methods on simulated data.** The bar chart displays the clustering performance of SpaAlign and other methods on the simulated dataset, evaluated using Mutual Information (MI), Normalized Mutual Information (NMI), Adjusted Mutual Information (AMI), Fowlkes-Mallows Index (FMI), Adjusted Rand Index (ARI), V-Measure, and Completeness.

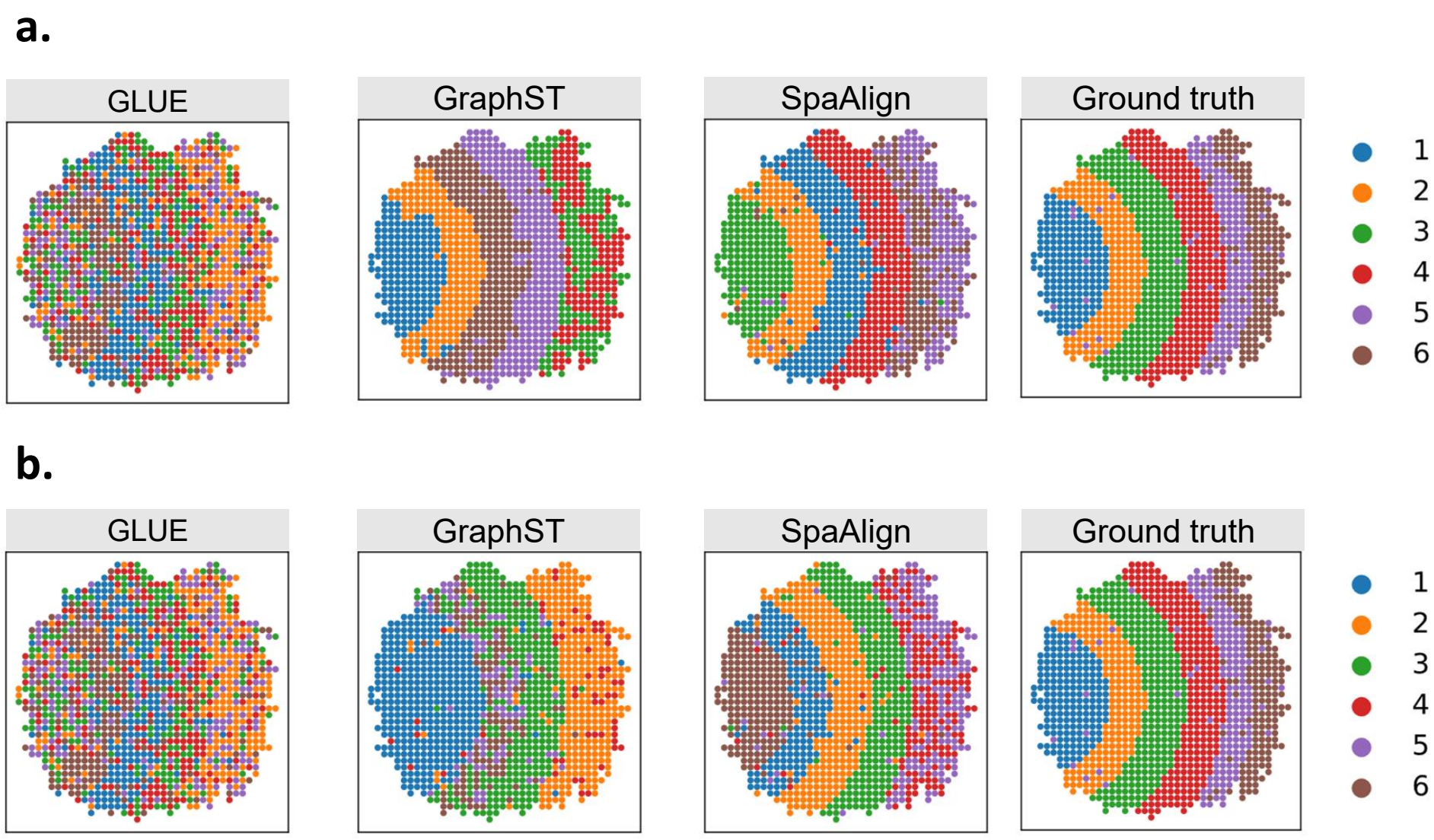

Fig. S3: **Ground-truth annotations and clustering results of the simulated dataset obtained by GLUE, GraphST, and SpaAlign. a,** Clustering results under intrinsic noise level = 0.2. **b,** Clustering results under intrinsic noise level = 1.0.

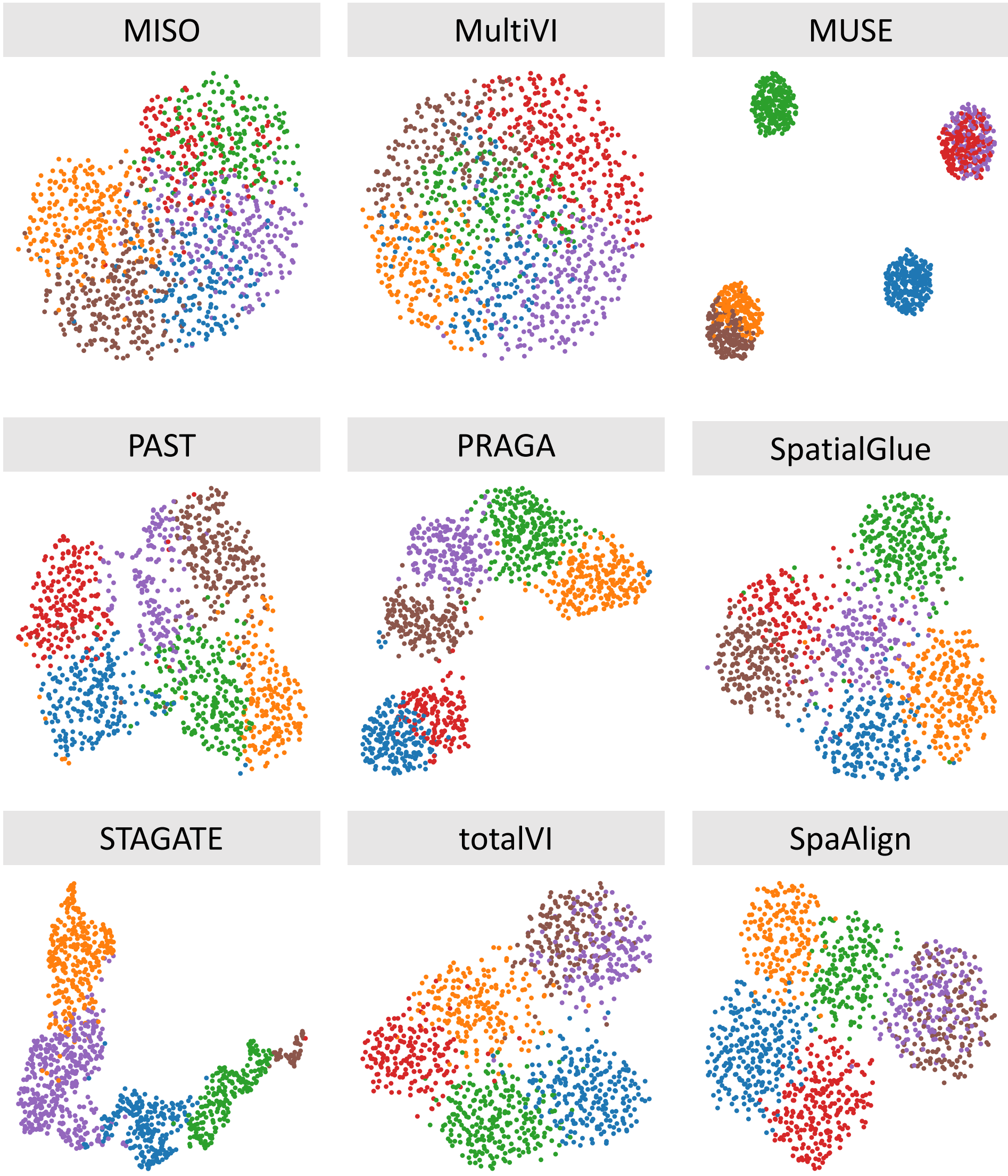

Fig. S4: **UMAP visualization of learned representations on the low-noise (0.2) simulated dataset.** 2D UMAP projections of latent space embeddings from SpaAlign and other baseline methods on the low-noise simulated data are presented. Colors represent six ground truth categories, allowing comparison of how methods separate the data.

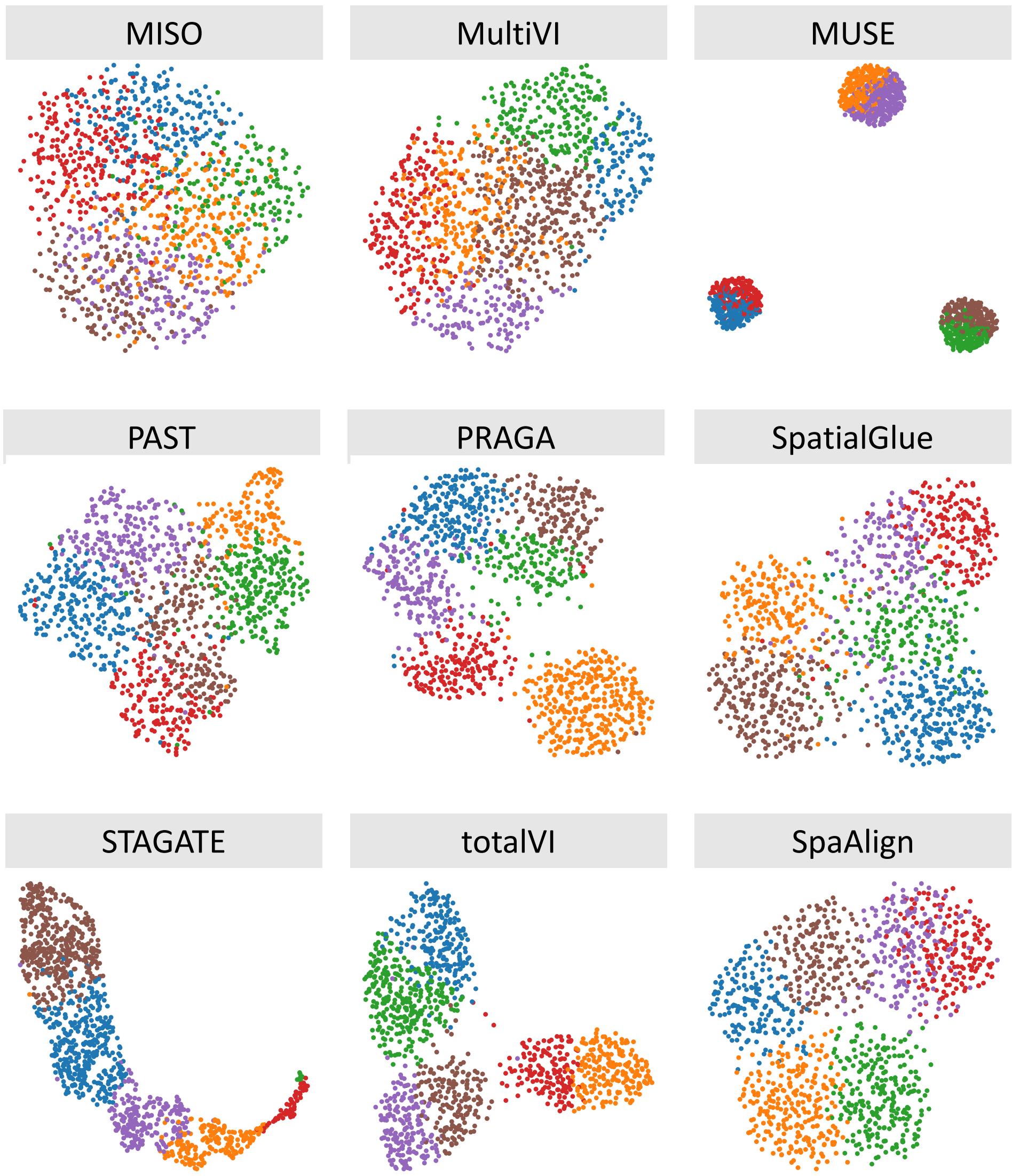

Fig. S5: **UMAP visualization of learned representations on the high-noise (1.0) simulated dataset.** 2D UMAP projections of latent space embeddings from SpaAlign and other baseline methods on the high-noise simulated data are presented. Colors represent six ground truth categories, allowing comparison of how methods separate the data under noisy conditions.

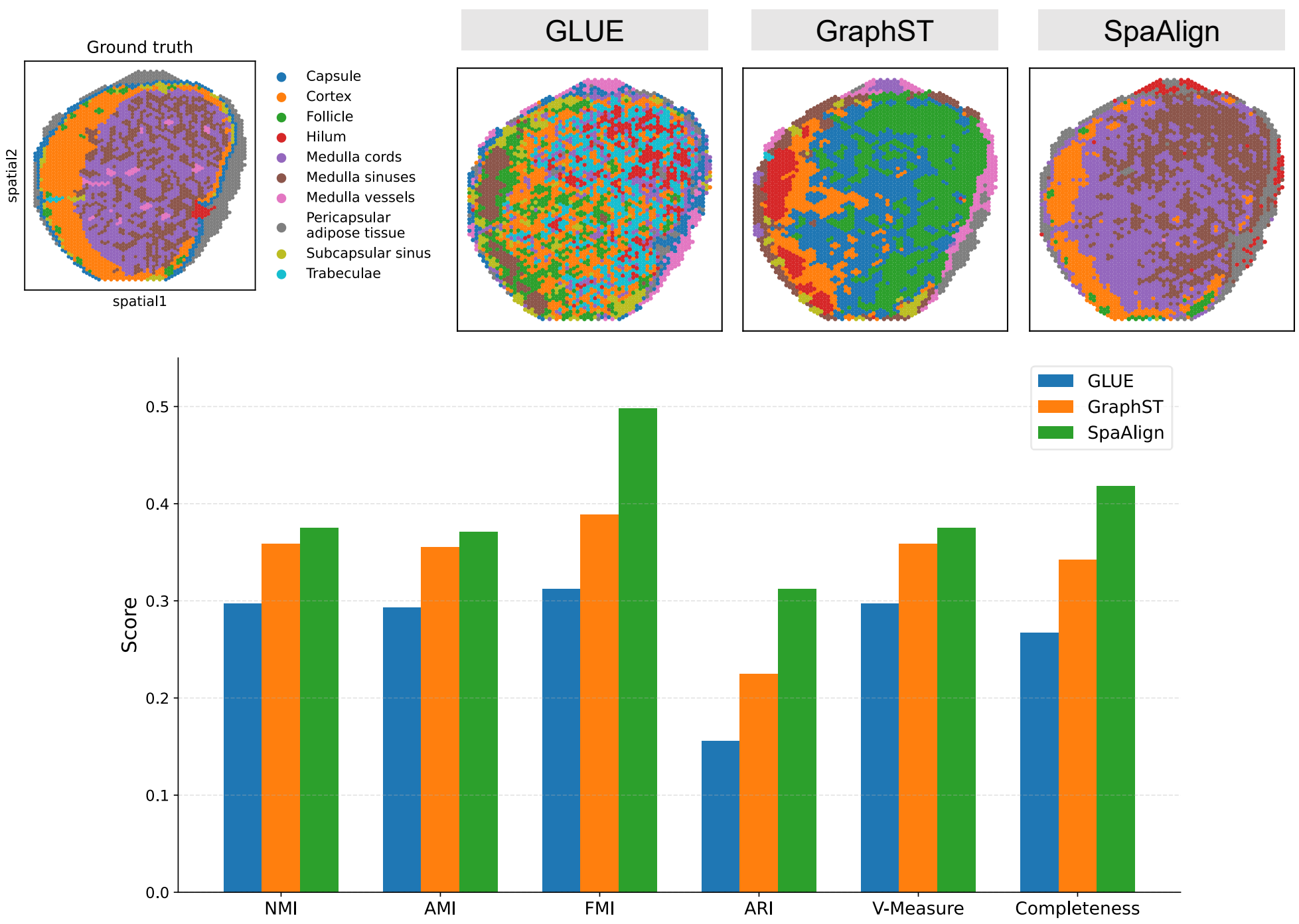

Fig. S6: **Quantitative benchmarking and clustering visualization of SpaAlign, GLUE, and GraphST on human lymph node dataset.**

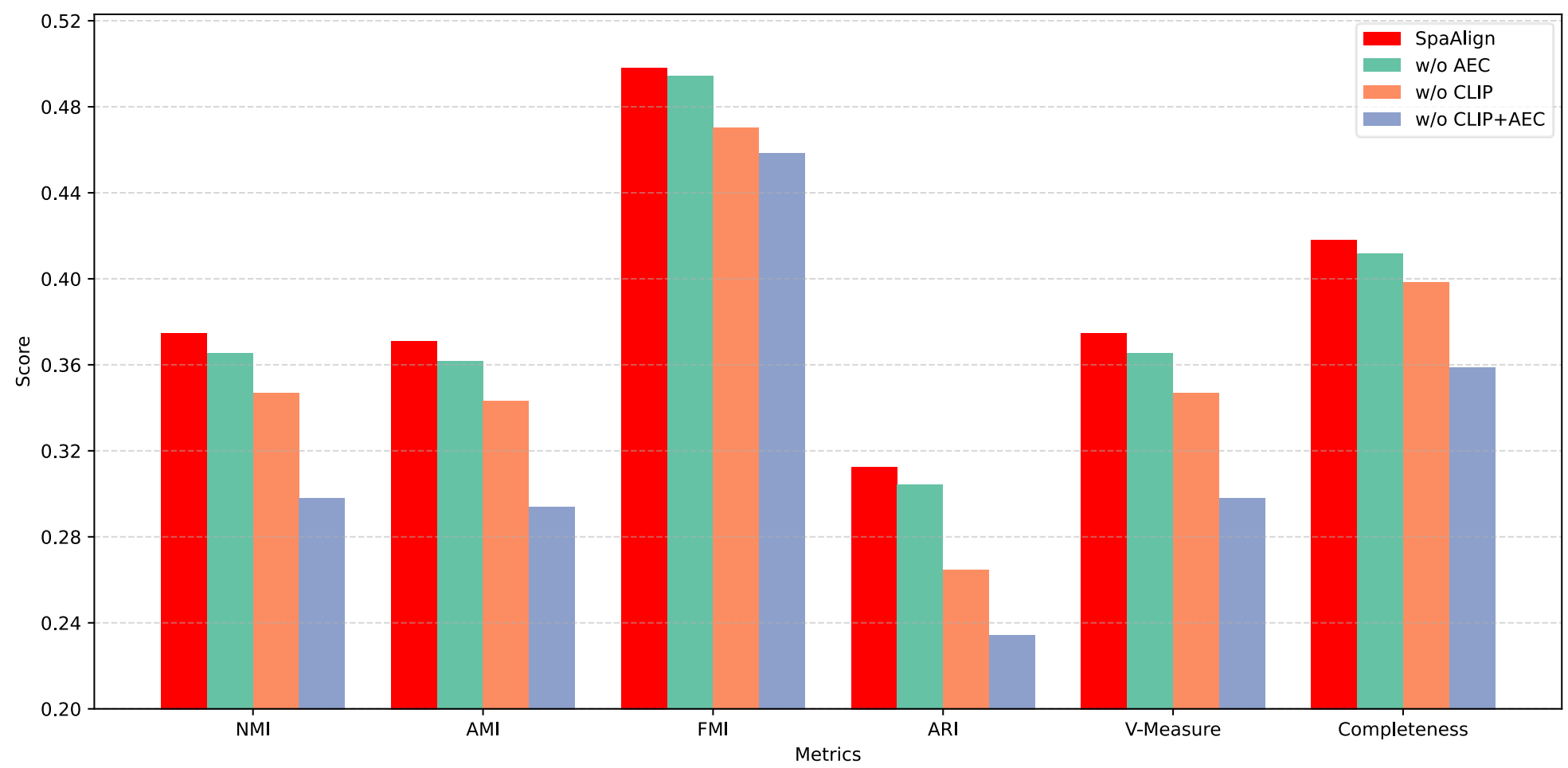

Fig. S7: **Ablation study of SpaAlign on human lymph node dataset.** Performance comparison across seven clustering evaluation metrics (MI, NMI, AMI, FMI, ARI, V-measure, and completeness) for the full SpaAlign model and its ablated variants: without the CLIP-based contrastive alignment module (w/o CLIP), without the AEC-based clustering refinement module (w/o AEC), and without both modules (w/o CLIP+AEC).

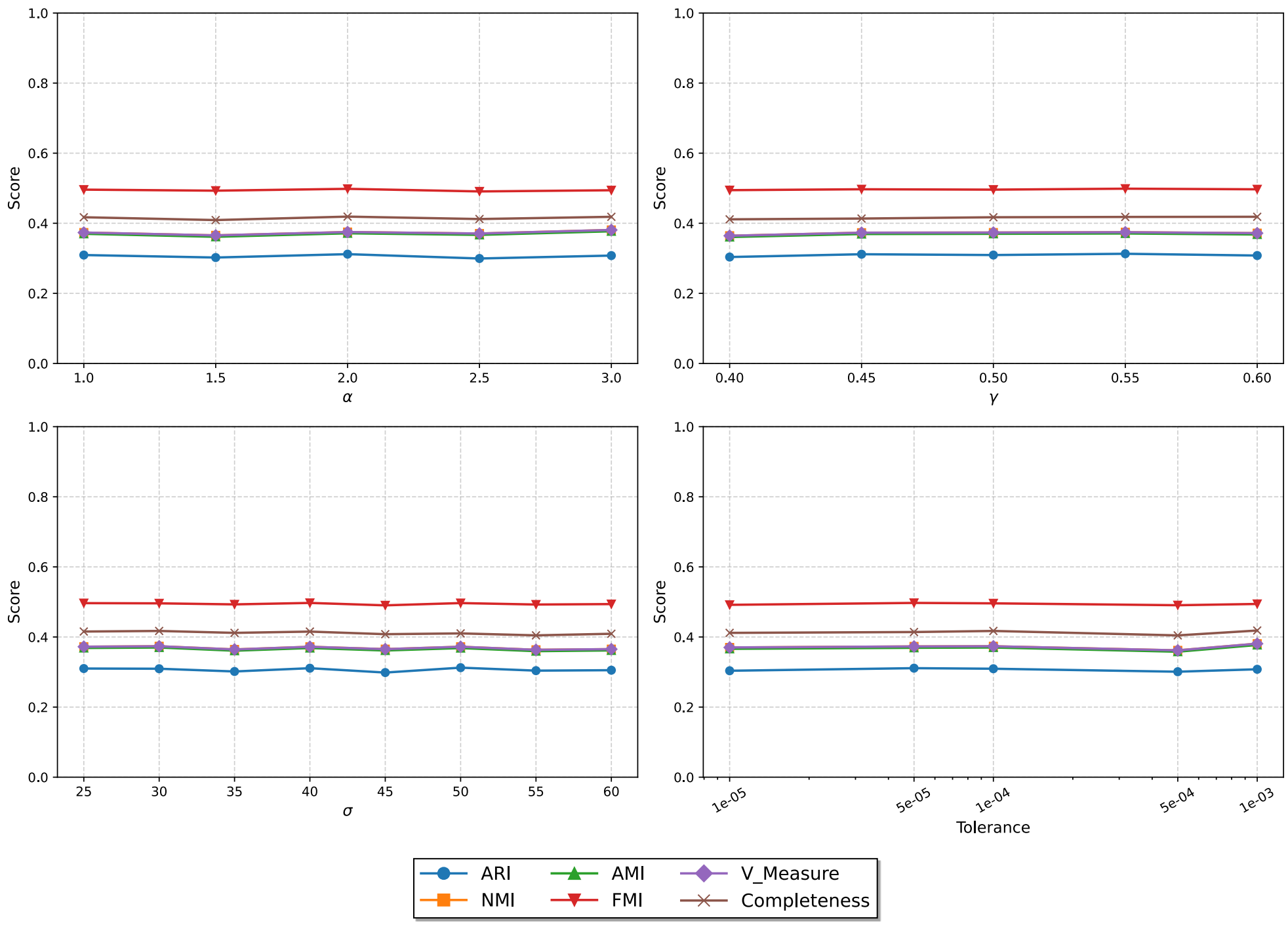

Fig. S8: **Hyperparameter sensitivity analysis on the HLN dataset.** Clustering performance across six metrics (ARI, NMI, AMI, FMI, V-measure, and Completeness) when varying α, γ, σ, and tol.

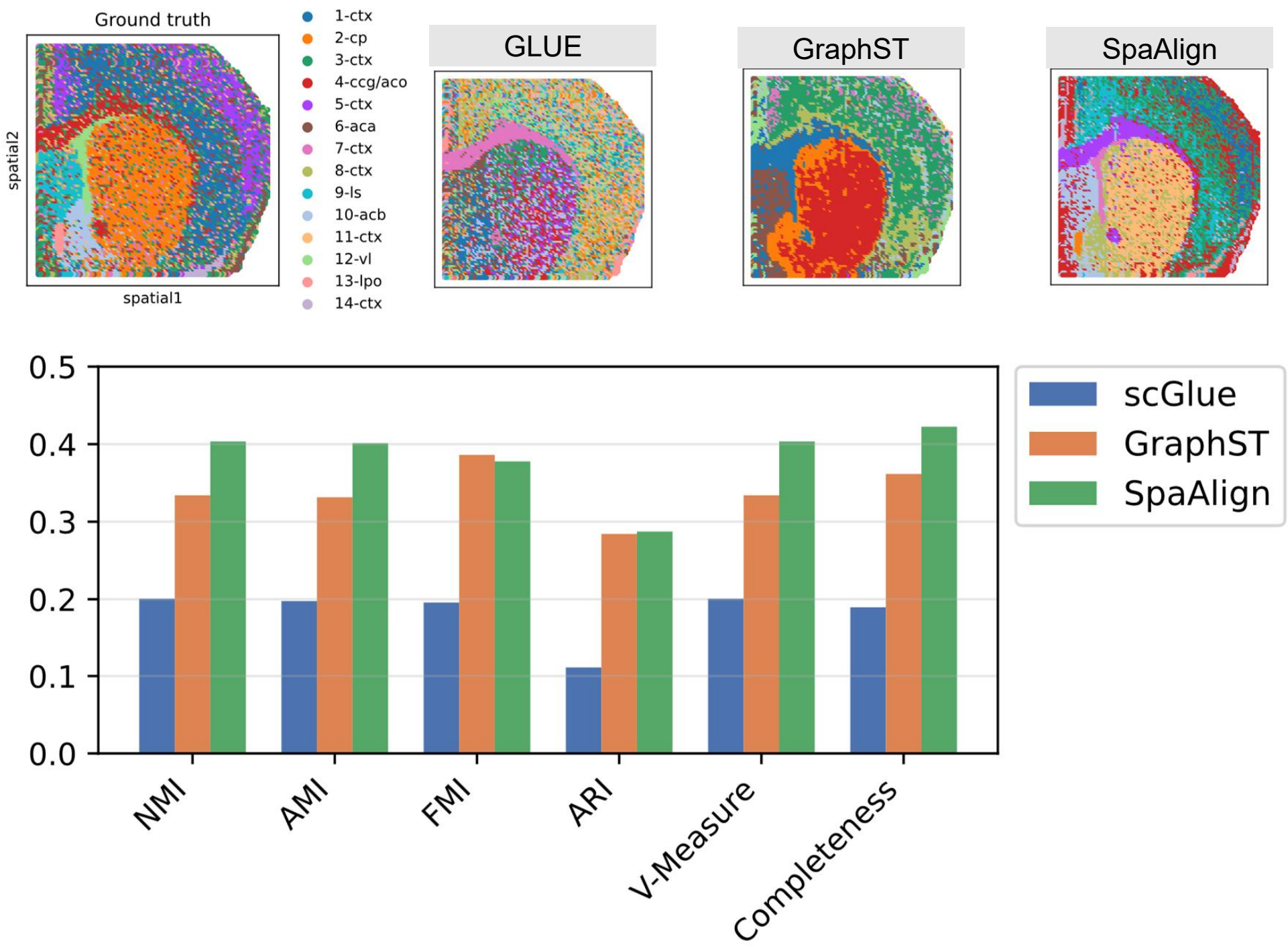

Fig. S9: **Quantitative benchmarking and clustering visualization of SpaAlign, GLUE, and GraphST on mouse brain data.**

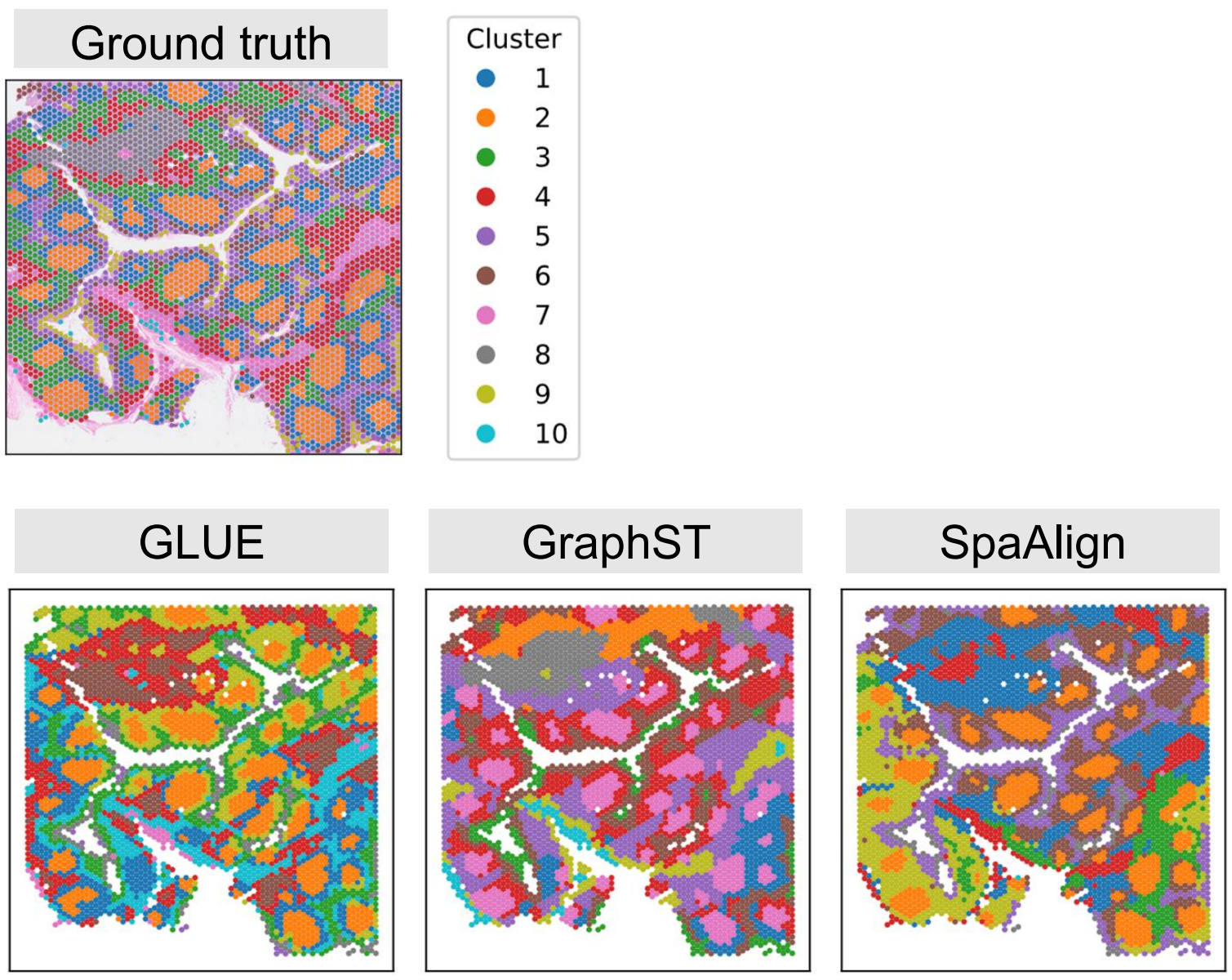

Fig. S10: **Clustering visualization of SpaAlign, GLUE, and GraphST on human tonsil data.**

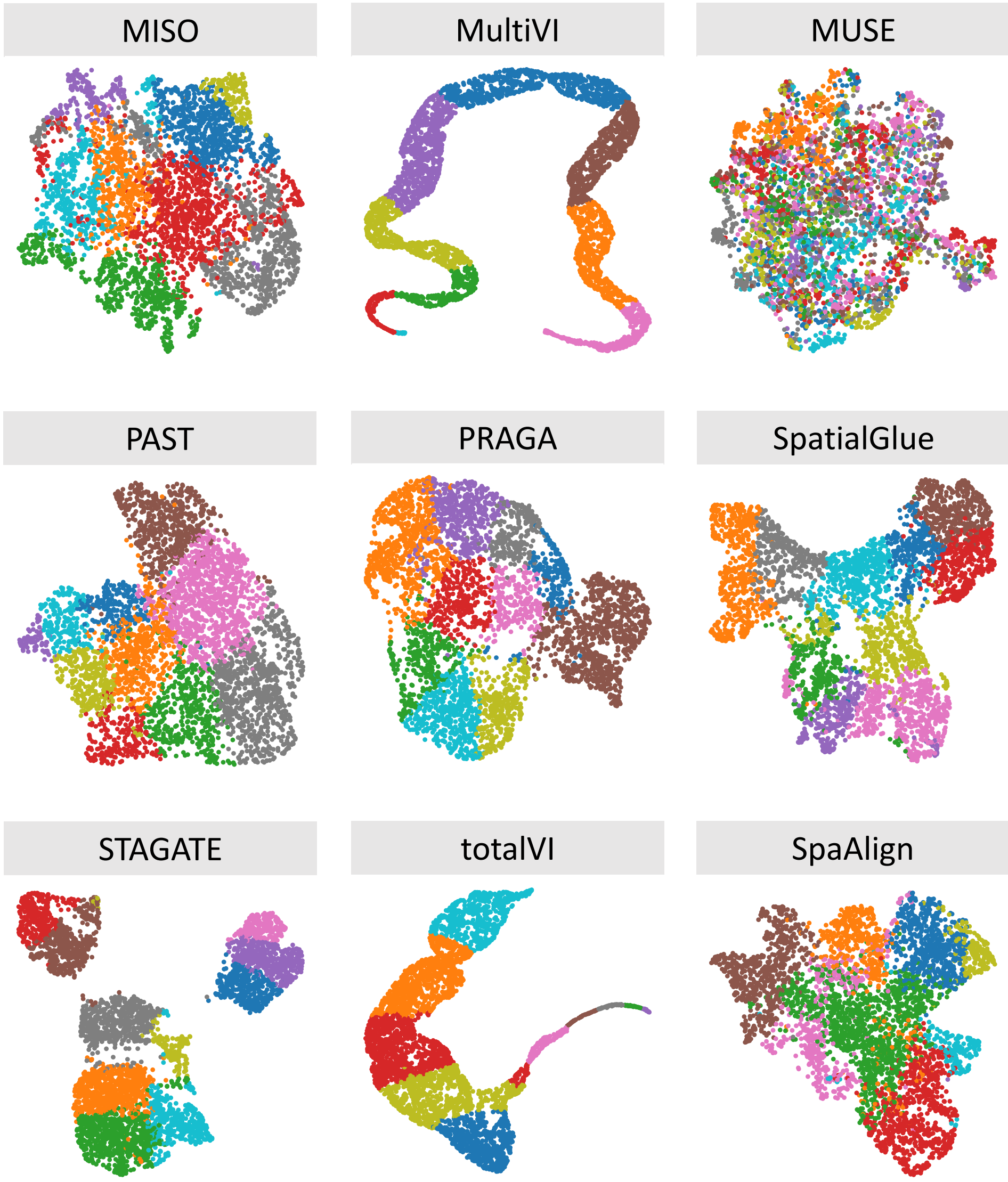

Fig. S11: **UMAP visualization of multi-modal integration on human tonsil data.** 2D UMAP projections of cell representations learned by SpaAlign and other baseline methods on a real-world human tonsil multi-modal dataset. Colors represent annotated spatial domains.

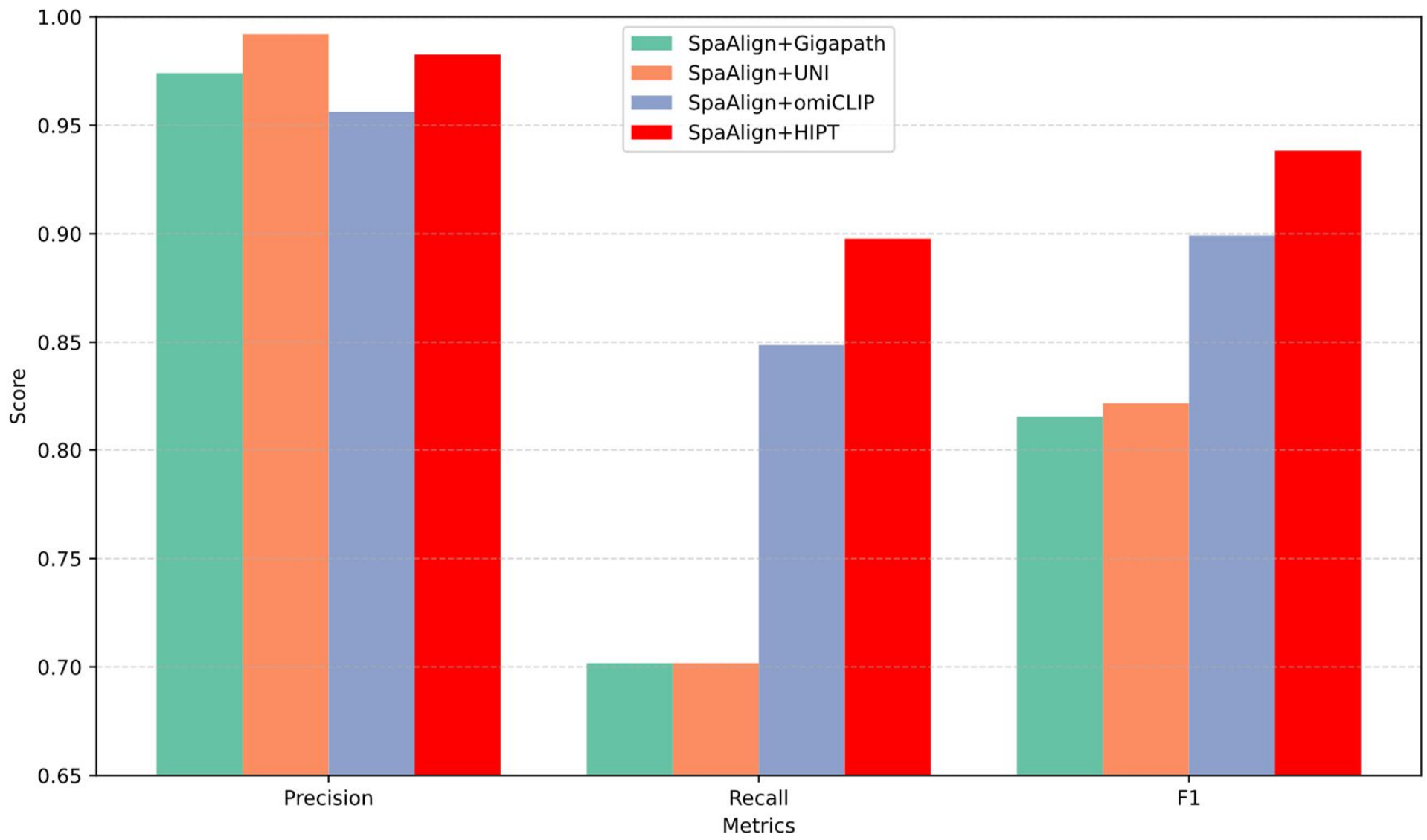

Fig. S12: **Ablation study on the impact of image encoders for Germinal Center identification.** This bar chart presents Precision, Recall, and F1 scores for Germinal Center identification on the human tonsil dataset. The SpaAlign framework was evaluated using image features from four pre-trained models: Prov-GigaPath, UNI, omiCLIP, and HIPT.

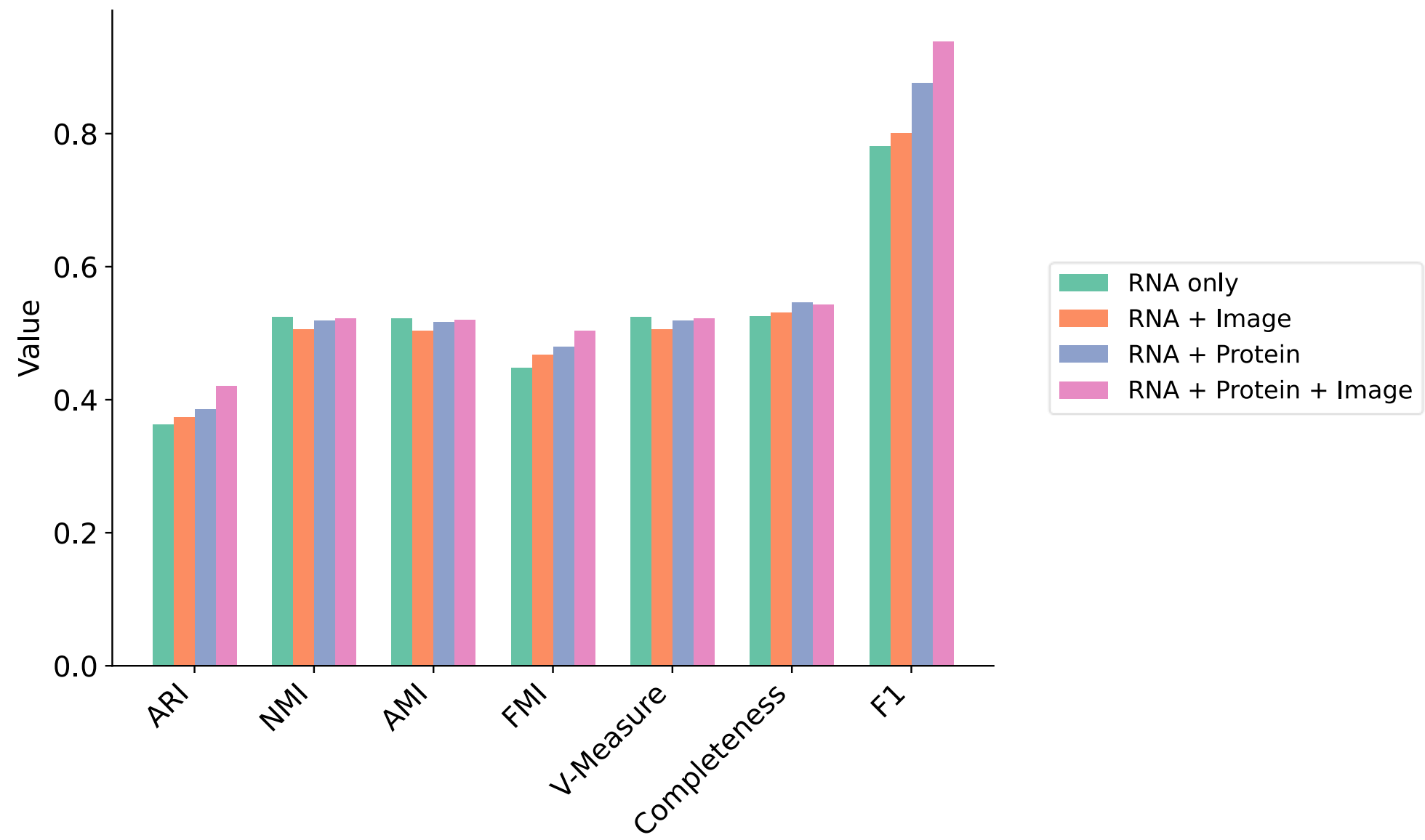

Fig. S13: **Ablation analysis of modality integration on the human tonsil data.**

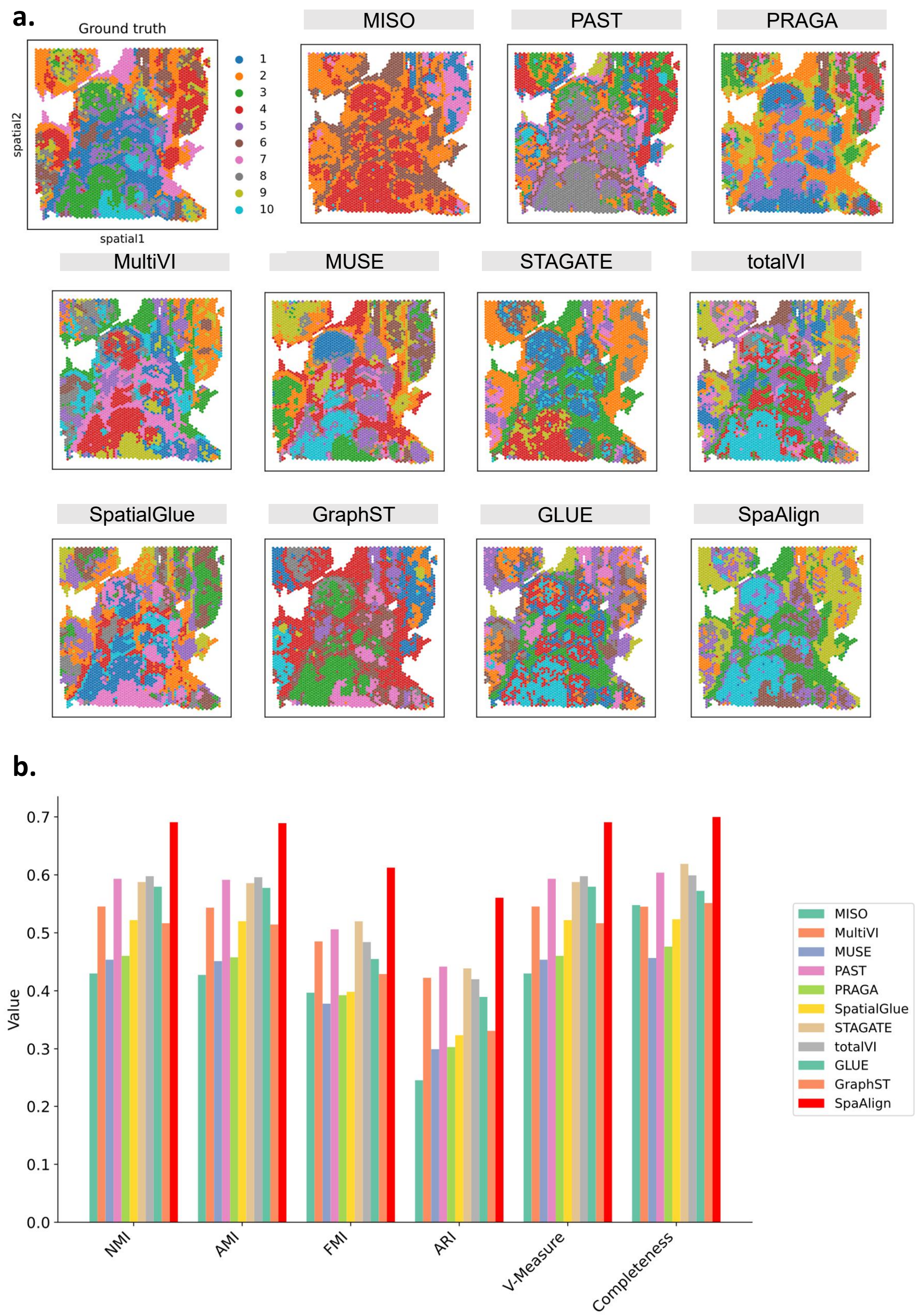

Fig. S14: **Quantitative benchmarking and clustering visualization of SpaAlign and competing methods on Human Breast Cancer spatial tri-modal data.** a, Clustering outputs from all evaluated methods on the Human Breast Cancer dataset. b, Quantitative comparison of six supervised metrics (NMI, AMI, FMI, ARI, V-Measure, and Completeness) across all methods.
